# Natural product biosynthetic potential reflects macroevolutionary diversification within a widely distributed bacterial taxon

**DOI:** 10.1128/msystems.00643-23

**Published:** 2023-11-29

**Authors:** Sandra Godinho Silva, Masun Nabhan Homsi, Tina Keller-Costa, Ulisses Rocha, Rodrigo Costa

**Affiliations:** 1 Department of Bioengineering, Instituto Superior Técnico, University of Lisbon, Lisbon, Portugal; 2 iBB–Institute for Bioengineering and Biosciences and i4HB–Institute for Health and Bioeconomy, Instituto Superior Técnico, University of Lisbon, Lisbon, Portugal; 3 Department of Molecular Systems Biology, Helmholtz Centre for Environmental Research–UFZ, Leipzig, Germany; 4 Department of Environmental Microbiology, Helmholtz Centre for Environmental Research–UFZ, Leipzig, Germany; Wageningen University, Wageningen, Netherlands

**Keywords:** biosynthetic gene clusters, carotenoids, comparative genomics, *Flavobacteriaceae*, machine learning, metagenome-assembled genomes, natural products

## Abstract

*Flavobacteriaceae* spp. are key players in global biogeochemical cycling and are known for their versatile carbohydrate and peptide catabolism. However, it is currently unknown whether secondary metabolism traits underlie their broad range of occurrence across the earth’s biomes. We examined 2,680 genomes to unveil an unprecedented phylogenetic signal dictating natural product biosynthesis diversification within the *Flavobacteriaceae* family. The distribution of secondary metabolite biosynthetic gene clusters (BGCs) across genomes usually follows macroevolutionary, genus-specific patterns. Noticeably, 88.6% of the observed BGCs were inferred to lead to the biosynthesis of likely novel natural products. We found an unanticipated, large diversity of taxon-specific BGCs encoding carotenoid and flexirubin pigments, the vast majority of which awaiting formal description. In particular, *Aquimarina* and *Kordia* spp. possessed large genomes, versatile catabolic traits, and a repertoire of BGCs possibly encoding drug-inspiring polyketides, non-ribosomal peptides, or post-translationally modified peptides. Using a machine learning approach (feature selection), we reveal that marine and non-marine *Flavobacteriaceae* genomes are differentially enriched in CAZymes and peptidases with distinct functionalities and molecular targets.

**IMPORTANCE:**

This is the most comprehensive study performed thus far on the biosynthetic potential within the *Flavobacteriaceae* family. Our findings reveal intertwined taxonomic and natural product biosynthesis diversification within the family. We posit that the carbohydrate, peptide, and secondary metabolism triad synergistically shaped the evolution of this keystone bacterial taxon, acting as major forces underpinning the broad host range and opportunistic-to-pathogenic behavior encompassed by species in the family. This study further breaks new ground for future research on select *Flavobacteriaceae* spp. as reservoirs of novel drug leads.

## INTRODUCTION

The family *Flavobacteriaceae* is a keystone taxon in terrestrial and marine biomes, being vastly acknowledged for its remarkable roles in the cycling of carbon worldwide (1). *Flavobacteriaceae* spp. are Gram-negative, mainly aerobic, non-spore-forming, and rod-shaped chemoorganotrophs (1). They constitute the largest family in the *Bacteroidota* phylum, with 151 validly published and correctly named genera as of June 2023 (https://lpsn.dsmz.de/family/flavobacteriaceae) (2). *Flavobacteriaceae* spp. have been isolated from a wide variety of environments, from coastal to oceanic and freshwater systems to soils and sediments and in association with humans, animals, and plants (3).

Such a broad range of occurrence and lifestyles reflects the high taxonomic diversity and genomic plasticity within the family. *Flavobacteriaceae* spp. have a rich repertoire of genes encoding carbohydrate-degrading enzymes (CAZymes) and peptidases, making them well equipped to utilize manifold carbon sources (4), which seems to contribute to their ability to thrive in diverse microniches. Recent genomic studies provided a glimpse into adaptive traits dictating *Flavobacteriaceae* evolution in, e.g., terrestrial vs marine biomes (5). However, we still lack an integral, holistic understanding of the coding potential and phylogenomic diversity of cultured and uncultured *Flavobacteriaceae* species in nature. In particular, current knowledge of natural product biosynthesis capacities and diversification among family members is scarce, limiting our ability to harness their metabolism and infer their adaptive features.

Using comparative genomics, we recently uncovered a previously unsuspected, natural product biosynthesis potential for the flavobacterial genus *Aquimarina* (6). The studied genomes were found to bear hundreds of secondary metabolite biosynthetic gene clusters (BGCs) involved in the production of drug-like candidates within several compound classes (6). These findings were intriguing because *Flavobacteriaceae* are best known for their primary roles in the breakdown of polysaccharides and peptides. Bioactive compounds described from *Flavobacteriaceae* spp. have shown antibacterial (7), antitumoral (8), cell growth promotion (9), and antioxidant and neuroprotective activities (10). Collectively, these early studies hint at a somewhat untapped reservoir of diverse bioactivities within the *Flavobacteriaceae* family. In fact, hundreds of underexplored species and genera in the family were not further investigated after their initial isolation and description (1). This can now be surpassed through the exploitation of recent developments in computational biology and the use of dedicated genome mining tools (11, 12).

In this study, we perform a large comparative genomic analysis of 1,923 *Flavobacteriaceae* genomes along with 757 genomes of the closest related *Weeksellaceae* family (outgroup) to (i) unveil the full secondary metabolite biosynthesis potential of the two most diverse families in the *Flavobacteriales* order, (ii) determine whether diversification of secondary metabolite biosynthetic gene clusters reflects the taxonomy of families and genera within the *Flavobacteriales* order, and (iii) identify genome signatures underlying secondary metabolism and catabolism of peptides and carbohydrates that reflect adaptation of *Flavobacteriaceae* spp. to terrestrial and marine biomes. We deeply explore the carbon- and peptidase-degrading capabilities within the *Flavobacteriaceae* family by coupling functional annotations of CAZymes and peptidases with a machine learning procedure (feature selection [FS]) to reveal the most distinguishing catalytic traits in marine vs non-marine habitats. Finally, we integrate results from BGC genome mining with CAZyme and peptidase annotations to shed light on the (natural product) biosynthetic and catabolic potential of *Flavobacteriaceae* species as interactive forces shaping the diversification and broad range of occurrence of a canonical microbial clade in nature.

## RESULTS

### Data set overview

We analyzed 2,680 genomes from the *Flavobacteriaceae* (1,923 genomes, 71.75%) and *Weeksellaceae* (757 genomes, 28.25%) families. The genomes were classified into 175 genera and 1,158 species using GTDB-Tk v.1.3.0. Following the quality parameters employed in this study, we identified 1,256 excellent-, 1,016 high-, and 408 medium-quality genomes in the data set (Fig. S1A tohrough C; Table S1), whose associated metadata were thoroughly recorded (Tables S2 and S3). Data on the geographic location, the employed sequencing technology, and the possible host association pertaining to the genomes analyzed in this study are provided in Fig. S1D to F.

**Fig 1:**
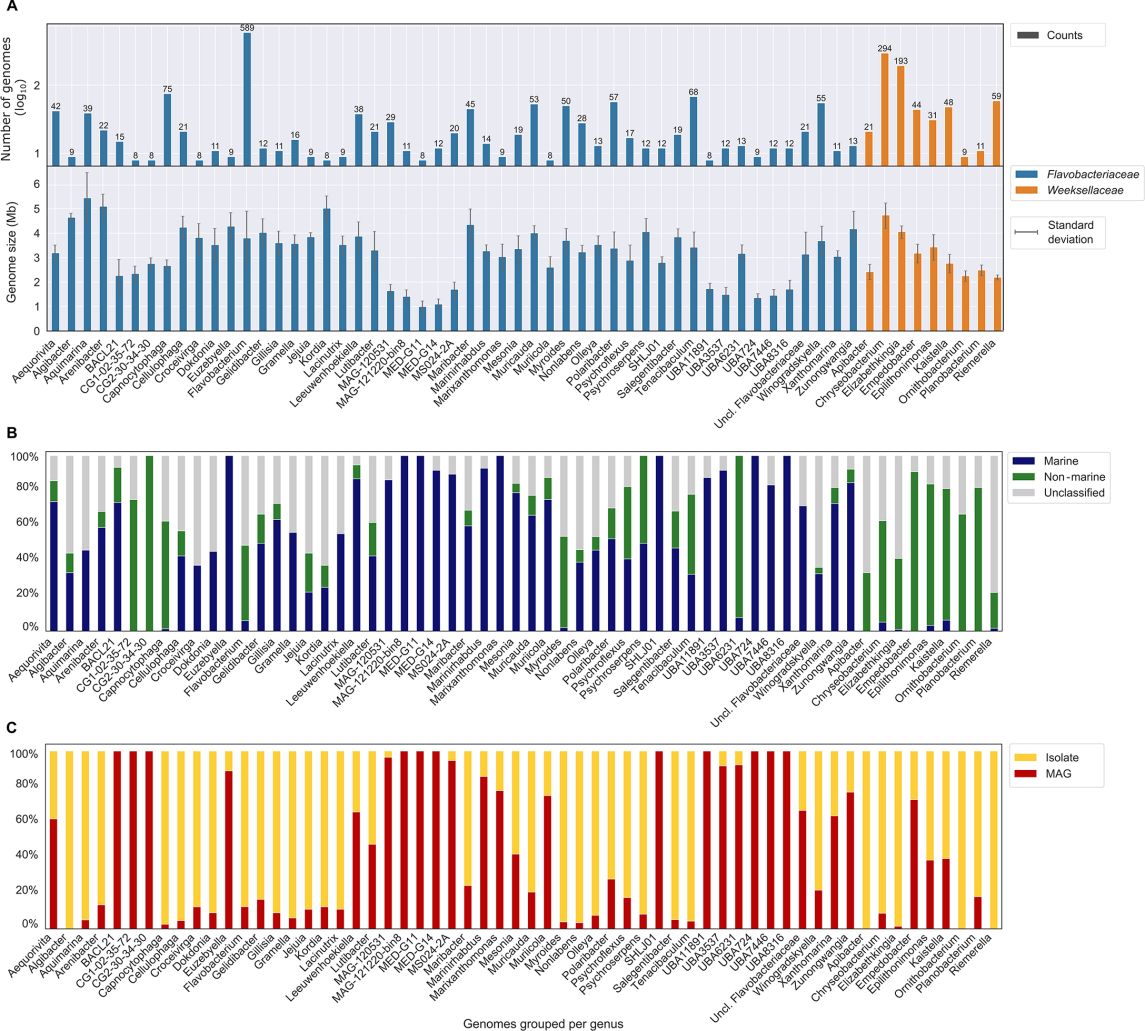
Basic genome features and provenance of the most representative genera analyzed in this study. Panels A to C display data for 60 (out of 175) *Flavobacteriaceae* and *Weeksellaceae* genera represented by at least eight genomes in the data set. (A) The total counts of genomes per genus (log_10_ scale) are shown along with the mean (±standard deviation) genome size per genus. Genera are grouped and colored by family: *Flavobacteriaceae* (blue) and *Weeksellaceae* (orange). (B) Proportion of genomes from marine, non-marine, or undetermined (“unclassified”) provenance within each genus. (C) Proportion of metagenome-assembled genomes (MAGs) and isolate genomes per genus.

*Flavobacterium* (*Flavobacteriaceae*) was by far the most frequent genus (589 genomes) in the data set, followed by the *Weeksellaceae* genera *Chryseobacterium* (294 genomes) and *Elizabethkingia* (193 genomes) (Fig. 1A). Across the entire data set, 792 genomes were classified as marine (29.55%) and 898 as non-marine (33.51%), and the remaining 990 genomes were left unclassified (36.94%) due to missing metadata and the existence of conflicting terms or because they were from transition environments (Fig. 1B; Tables S2 and S3).

Metagenome-assembled genomes (MAGs) represented around a quarter (*n* = 692) of the genomes analyzed (Fig. 1C; Fig. S1). On average, MAGs possessed smaller genome sizes (2.75 ± 1.05 Mb) than isolate genomes (3.87 ± 0.96 Mb) (Mann-Whitney *U* test, *P* < 0.0001). The same trend was observed for the mean number of open reading frames (ORFs) per genome (Mann-Whitney *U* test, *P* < 0.0001), with MAGs and isolate genomes possessing 2,499 (±954) and 3,444 (±826) ORFs on average. Most of the MAGs (63.4%) were of marine origin, while the remaining were classified as non-marine (28.3%) or left unclassified (8.2%). In contrast, a higher proportion (46.9%) of isolate genomes remained unclassified in terms of origin. In total, 298 genomes (11.12%) were not assigned to formally described genera, most of which were MAGs (Fig. S2).

### Genome diversity and relatedness among *Flavobacteriaceae* and *Weeksellaceae* species

**Fig 2:**
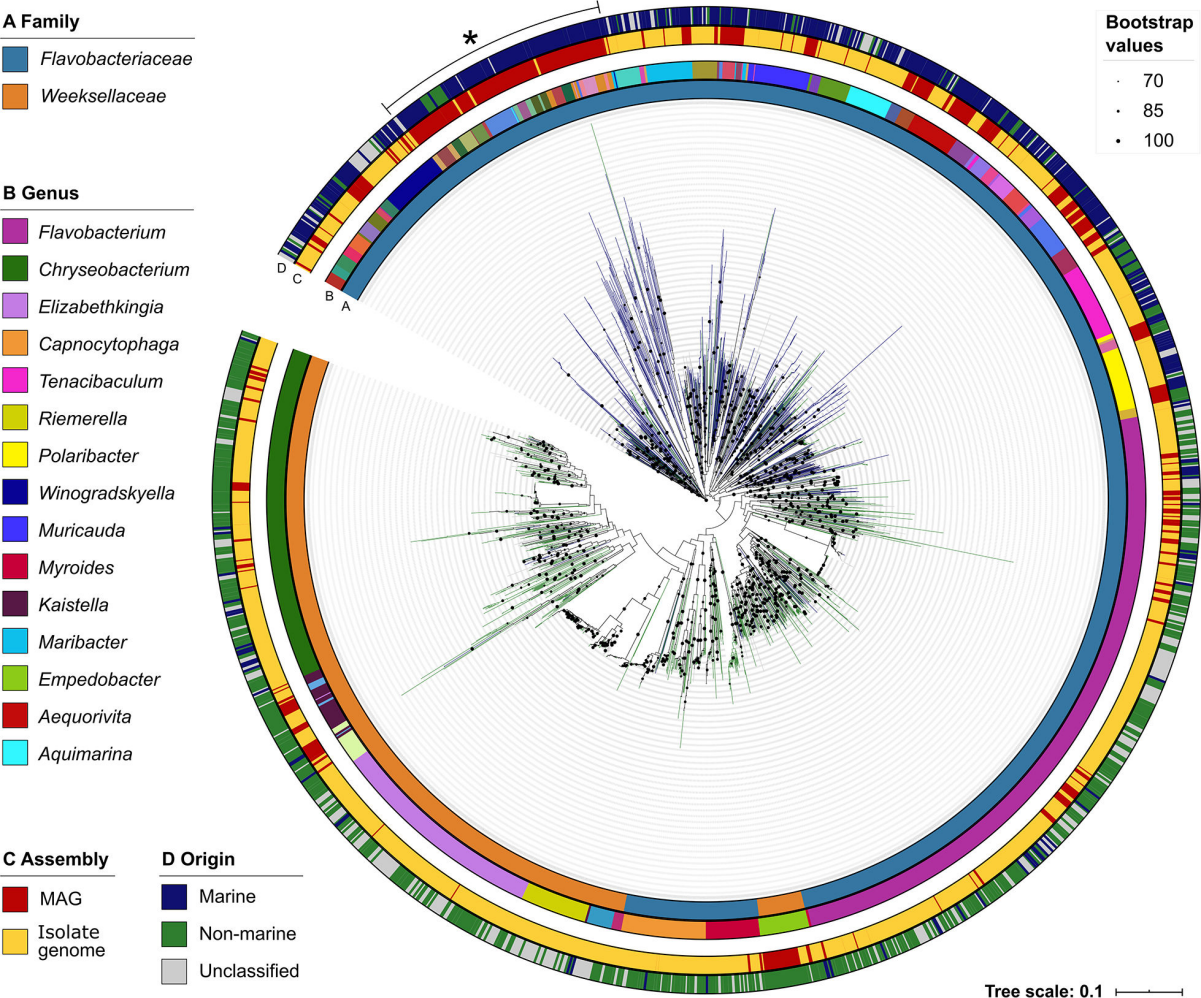
Genome-wide cladogram of *Flavobacteriaceae* and *Weeksellaceae* species. The cladogram is based on the pairwise distance between genome sketches composed of the top k-mers, created and sorted with the MinHash algorithm, and constructed with the neighbor-joining method (13). The tree is drawn to scale and represents the k-mer-based similarities of 2,359 genomes across 60 genera possessing ≥8 genomes in the data set (see Fig. 1 for details). Black dots on clade nodes represent confidence values >70% estimated by bootstrapping (100 repetitions). The asterisk symbol highlights a group of genotypically related MAGs within the *Flavobacteriaceae* family. Rings A (innermost ring) to D (outermost ring) present basic organismal (genome) features as follows. (A) Taxonomic classification at the family level (*Flavobacteriaceae* or *Weeksellaceae*). (B) Taxonomic classification at the genus level [for the sake of simplicity, the legend on the left side of the cladogram displays the names of the 15 most abundant genera (out of 60 genera colored in the ring)]. (C) Genome type (MAGs or isolate genomes). (D) Genome origin (marine, non-marine, or unclassified). Tree leaves are colored blue (marine), green (non-marine), or gray (unclassified) according to genome origin. Family- and genus-level taxonomic assignments were obtained with GTDB-Tk, which employs a phylogenomics-based framework for taxonomic classification.

**Fig 3:**
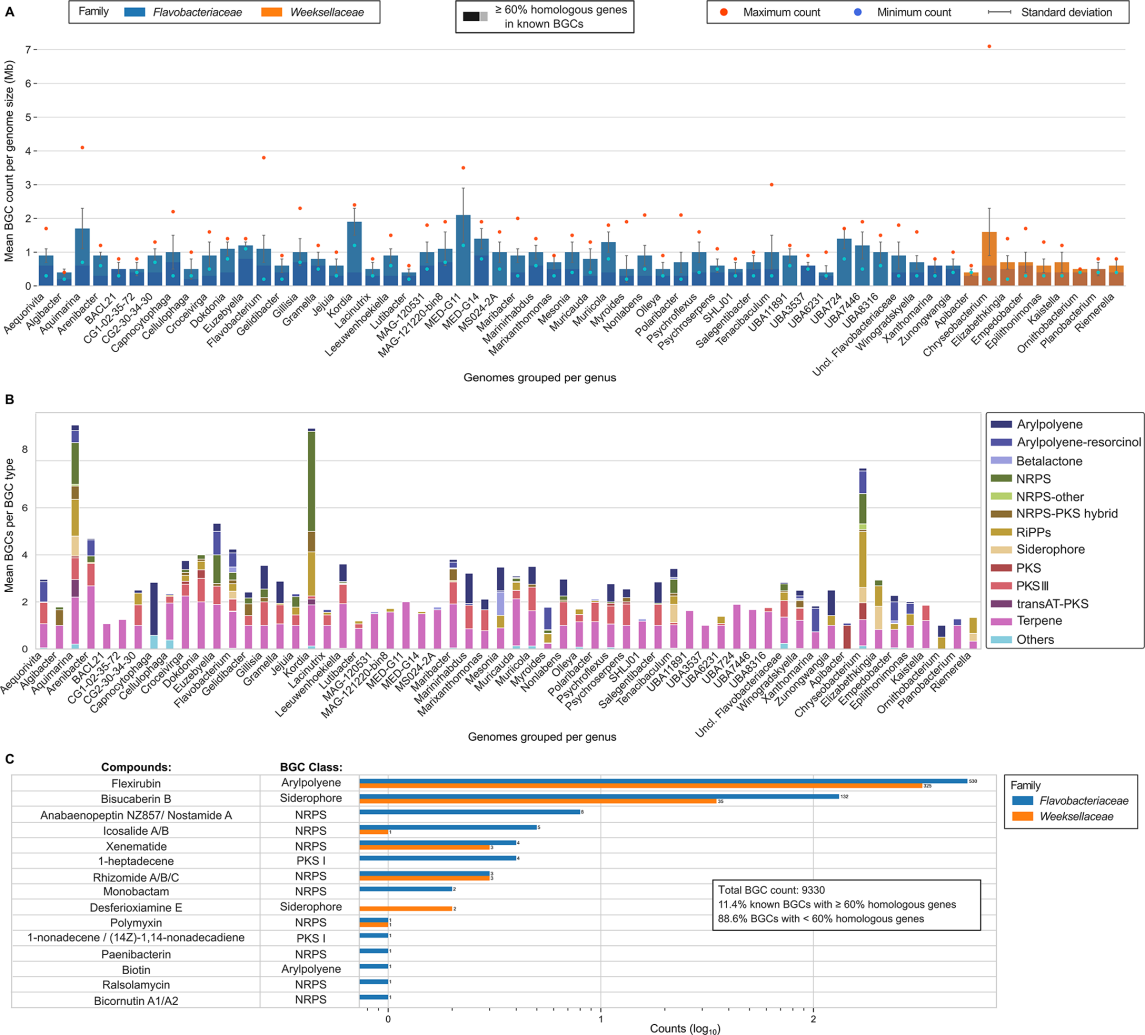
Genomics-aided inference of secondary metabolism profiles. Overview of the identified BGCs and their correspondence with the Minimum Information about a Biosynthetic Gene cluster (MIBiG) database across 60 genera represented by at least eight genomes (Fig. 1). (A) Mean BGC count per genus normalized by genome size (Mb). Genera are grouped and colored by family: *Flavobacteriaceae* (blue) and *Weeksellaceae* (orange). Darker shades in each bar represent the mean number of BGCs displaying ≥60% homologous genes with MIBiG BGCs within each genus. The standard deviation and the minimum (blue dots) and maximum (red dots) BGC counts per genus are displayed. (B) Mean number of BGCs per BGC type and per genus. Each color in the bar chart represents a different BGC compound class. This figure shows only compound classes represented by more than 100 BGCs in the whole data set. The remaining compound classes were included in the “others” category. (C) Number of BGCs identified in the data set presenting ≥60% homologous genes with MIBiG BGCs. The column on the left lists all compounds in the MIBiG database putatively encoded by at least one BGC from our data set, considering the minimum 60% homology threshold to known BGCs employed in this study.

Our whole genome sequence assessment encompassed 2,359 genomes (80.0% of the total number of genomes in the data set) across two families and 60 genera, with each genus represented by at least eight genomes (Fig. 2). As such, this assessment provides an overview of the genomic diversification across a select group of target genera with replicate genomes (*n* ≥ 8), which was used in this study to display the major trends in the data in a tractable fashion (see also Fig. 1, 3, and 7). Except for *Empedobacter*, the placement of all genera in the cladogram was coherent with their classification at the family level. By connecting genome taxonomy (using GTDB-Tk) with manually curated metadata, we could ascertain the prevailing terrestrial nature of the *Weeksellaceae* family in contrast to the *Flavobacteriaceae* family, which contained genera found to be exclusively/predominantly marine (e.g., *Aquimarina*, *Euzebyella*, and *Marinirhabdus*) and exclusively/predominantly terrestrial (e.g., *Capnocytophaga*, *Flavobacterium*, and *Myroides*) or to possess equitable amounts of genomes from both terrestrial and marine origins (e.g., *Psychroflexus* and *Psychroserpens*) (Fig. 2; see also Fig. 1A and B). Of the 564 MAGs portrayed in Fig. 2, 173 MAGs (30.7%) were found to form diverse, deeply branching yet genotypically related clades within the *Flavobacteriaceae* family, while the remaining MAGs (*n* = 391, 69.3%) were, altogether, spread over most of the major branches of the tree (Fig. 2). We observed that placement of bacterial genera across the cladogram, which was built using the neighbor-joining method on a matrix of k-mer profile similarities between the genomes (see Materials and Methods and the legend to Fig. 2 for details), was overall congruent with the genus-level taxonomic assignment of these genomes with GTDB-Tk, which uses a highly sensitive phylogenomic framework based on the alignment of single-copy marker genes (Fig. 2).

### Natural product biosynthesis potential: general features

We uncovered 9,330 BGCs from 2,680 bacterial genomes, delivering new insights into the genetic space underlying natural product biosynthesis within the *Flavobacteriaceae* (6,167 BGCs) and *Weeksellaceae* (3,163 BGCs) families. Two cultivated genera and one uncultivated genus in the *Flavobacteriaceae* family, *Aquimarina* and *Kordia* and MED-G11, respectively, stood out regarding the average number of BGCs present per genome size (Fig. 3A). However, when absolute BGC counts per genome were recorded (Fig. S3), the genera *Aquimarina*, *Kordia* (*Flavobacteriaceae*), and *Chryseobacterium* (*Weeksellaceae*) were found to display the highest values across the data. Each of these three genera displayed significantly higher BGC counts per genome size than all the remaining genera combined as a single group (Mann-Whitney *U* test, *P* < 0.0001). Even though MAGs presented, on average, less BGCs in absolute numbers than isolate genomes, when normalized by genome size, no significant difference in BGC counts between the two groups was observed (Mann-Whitney *U* test, *P* = 0.4).

The most frequent compound types predicted with antiSMASH to be encoded by BGCs were carotenoids (2,117 BGCs, none of which possessing ≥60% homologous genes with the Minimum Information about a Biosynthetic Gene cluster [MIBiG] BGCs) and flexirubins (1,234 BGCs, of which 855 [69.3%]) had ≥60% of genes displaying homology with those from known BGCs; Table S4). BGCs found in this study were estimated to encode metabolites or enzymes of the following types: terpenes (including carotenoids, 30.53%), “others” (this BGC “type” includes the pigment flexirubin, 18.50%), ribosomally synthesized and post-translationally modified peptides (RiPPs, 12.94%), type III polyketide synthases (T3-PKSs, 10.74%), non-ribosomal peptide synthetases (NRPSs, 8.33%), NRPS-PKS hybrids (6.68%), siderophores (6.49%), other PKSs, including type I PKSs (3.16%), other NRPSs (2.13%), and *trans*-AT PKS (0.50%). Only four genera displayed *trans*-AT PKS BGCs: *Aquimarina* (where 7.8% of the identified BGCs were from this class), *Kordia* (2.6%), *Flavobacterium* (0.5%), and the unclassified marine *Flavobacteriaceae* genus SCGC-AAA160-P02 (13.3%), only possessing four genomes (Fig. 3B; see Table S1 for details).

### Insights into biosynthetic novelty

Only 11.4% (1,064) of all BGCs shared ≥60% homologous genes with those from BGCs present in the MIBiG database (14) and have been categorized here as BGCs putatively encoding known (100% homology) or close-to-known compounds (Fig. 3C; Table S4). Such BGCs were present on isolate genomes more often, and most were found to potentially code for flexirubins (855 BGCs—as mentioned above—possessing 60% to 97% homologous genes with MIBiG entries) and for the siderophore bisucaberin B (167 BGCs showing 66% to 100% homologous genes with MIBiG entries). Within this group of BGCs, 103 BGCs possessed 100% homologous genes with MIBiG BGCs and were found to encode either bisucaberin B (63 BGCs) or diverse non-ribosomal peptides such as anabaenopeptin, xenematide, icosalide A/B, rhizomide, and the β-lactam antibiotic monobactam (for details, see File S1). A wealth of BGCs annotated by antiSMASH (*n* = 8,866) possessed <60% homologous genes with BGCs in the MIBiG database. These included BGCs possessing rather low proportions of homologous genes with established BGCs known to code for bacillomycin D (20%), colanic acid (9%–14%), ET-743 (trabectedin), polysaccharide B (6%–10%), bacillaene (14%–42%), zwittermicin A (7%–22%), phormidolide (28%), WLIP A, and eicosapentaenoic acid-like compounds (9%) (Table S4; see File S1 for details).

### Organization of BGCs into gene cluster clans and families

**Fig 4:**
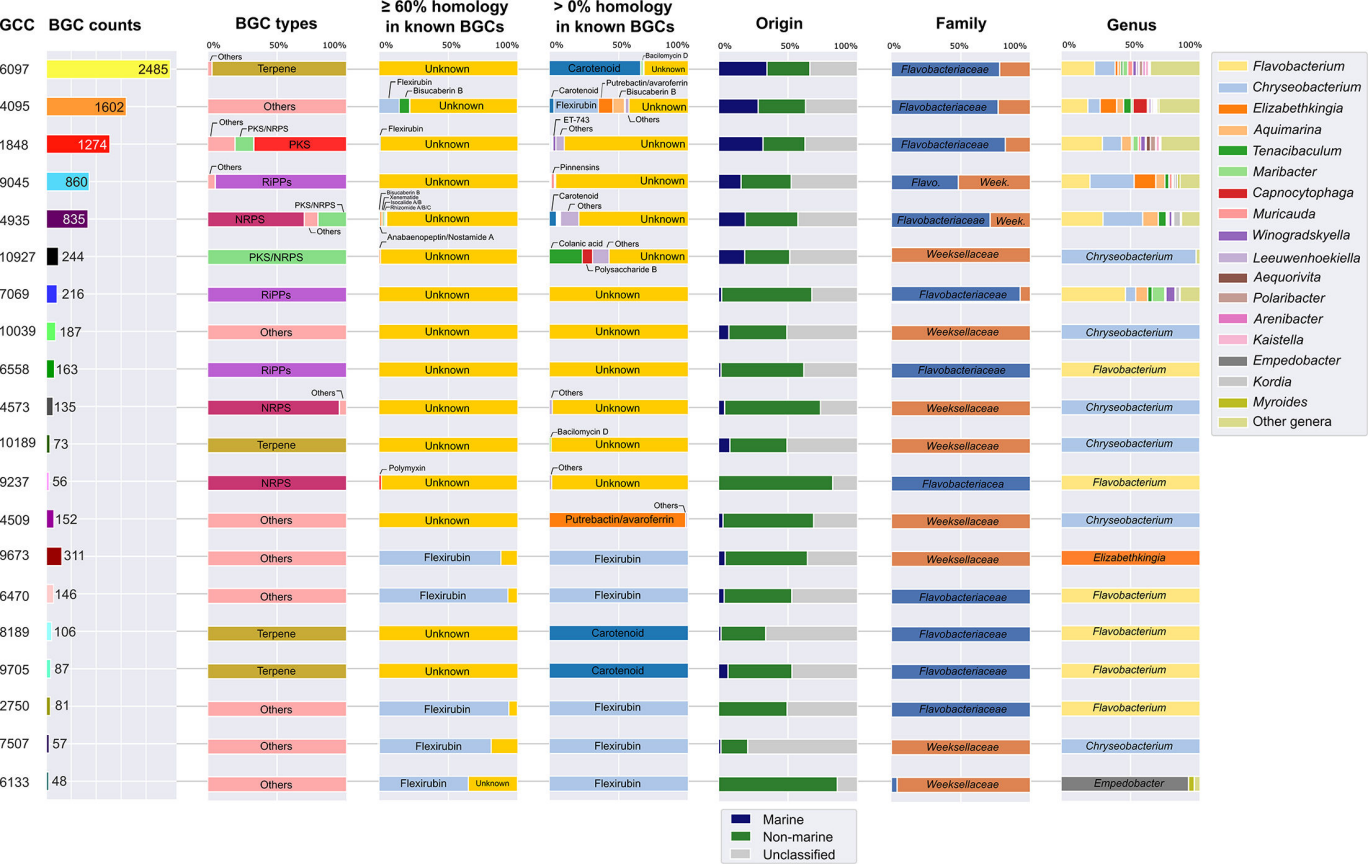
Description of the largest gene cluster clans (GCCs) in the data set. List of the top 20 largest GCCs. The first column displays the total number of BGCs per GCC. The following columns portray the relative abundance of BGCs within each clan for several features: proportion of BGCs putatively encoding major compound classes following the BiG-SCAPE classification scheme (“BGC types”), proportion of BGCs found in this study for which ≥60% or ≥0% homologous genes are present in MIBiG BGCs (“≥60%/0% homology in known BGCs”), genome/BGC origin, and family- and genus-level classification of the genomes hosting BGCs within each clan.

BGCs found in this study were grouped into 2,297 gene cluster families (GCFs) and 102 gene cluster clans (GCCs) using the Biosynthetic Gene Similarity Clustering and Prospecting Engine (BiG-SCAPE) (Table S5; see Materials and Methods for the grouping of BGCs into GCCs and GCFs). Figure 4 lists the 20 largest GCCs observed across the data. These GCCs contained from 48 (clan 6133) to as many as 2,485 (clan 6097) BGCs and were inspected in more detail regarding their putative biosynthetic novelty, the compound or enzyme classes they encode, and the provenance and taxonomy of their bearing genomes (Fig. 4). Although several such clans (12 in 20) were predicted to code for a major compound class (e.g., terpenes, polyketides, and non-ribosomal peptides), the majority were composed of BGCs sharing homologous genes with <60% of the genes from MIBiG BGCs. The exceptions were clans 9673, 6470, 2750, 7507, and 6133, all dominated by BGCs possessing high homology with flexirubin-encoding BGCs in the MIBiG database (Fig. 4). When stringency was relaxed to find MIBiG matches possessing any homologous genes with BGCs present in our top 20 GCCs, more GCCs could be assigned to the potential biosynthesis of specific compound/enzyme classes (Fig. 4). This was the case of GCCs 8189 and 9705, which were found to contain BGCs exclusively assigned to the biosynthesis of carotenoids, and GCC 4509, containing BGCs underlying the putative biosynthesis of the siderophore putrebactin/avaroferrin.

The more populous GCCs (that is, those containing the largest numbers of BGCs) were considered “generalist clans,” as they were often composed of BGCs of both marine and non-marine origins belonging to diverse genera in both the *Flavobacteriaceae* and *Weeksellaceae* families (e.g., GCCs 6097, 4095, 1848, 9045, 4935, and 7069). However, the majority (*n* = 14) of the top 20 GCCs can be considered “specialist clans,” as they were usually associated with a prevailing origin (most often terrestrial) and presented a high taxonomic fidelity at both the family and genus levels. That was the case of terpene-encoding (including putative carotenoid-encoding) clans 10189, 8189, and 9705, which contained BGCs exclusively found in the genera *Chryseobacterium* (clan 10189, family *Weeksellaceae*) and *Flavobacterium* (clans 8189 and 9705, family *Flavobacteriaceae*), the NRPS-encoding clans 4573 (*Chryseobacterium* and *Weeksellaceae*) and 9237 (*Flavobacterium* and *Flavobacteriaceae*), the RiPP-encoding clan 6558 (*Flavobacterium*), and several flexirubin-encoding clans exclusive to the genera *Elizabethkingia*, *Chryseobacterium*, *Empedobacter,* and *Flavobacterium* (Fig. 4).

### BGC networks

**Fig 5:**
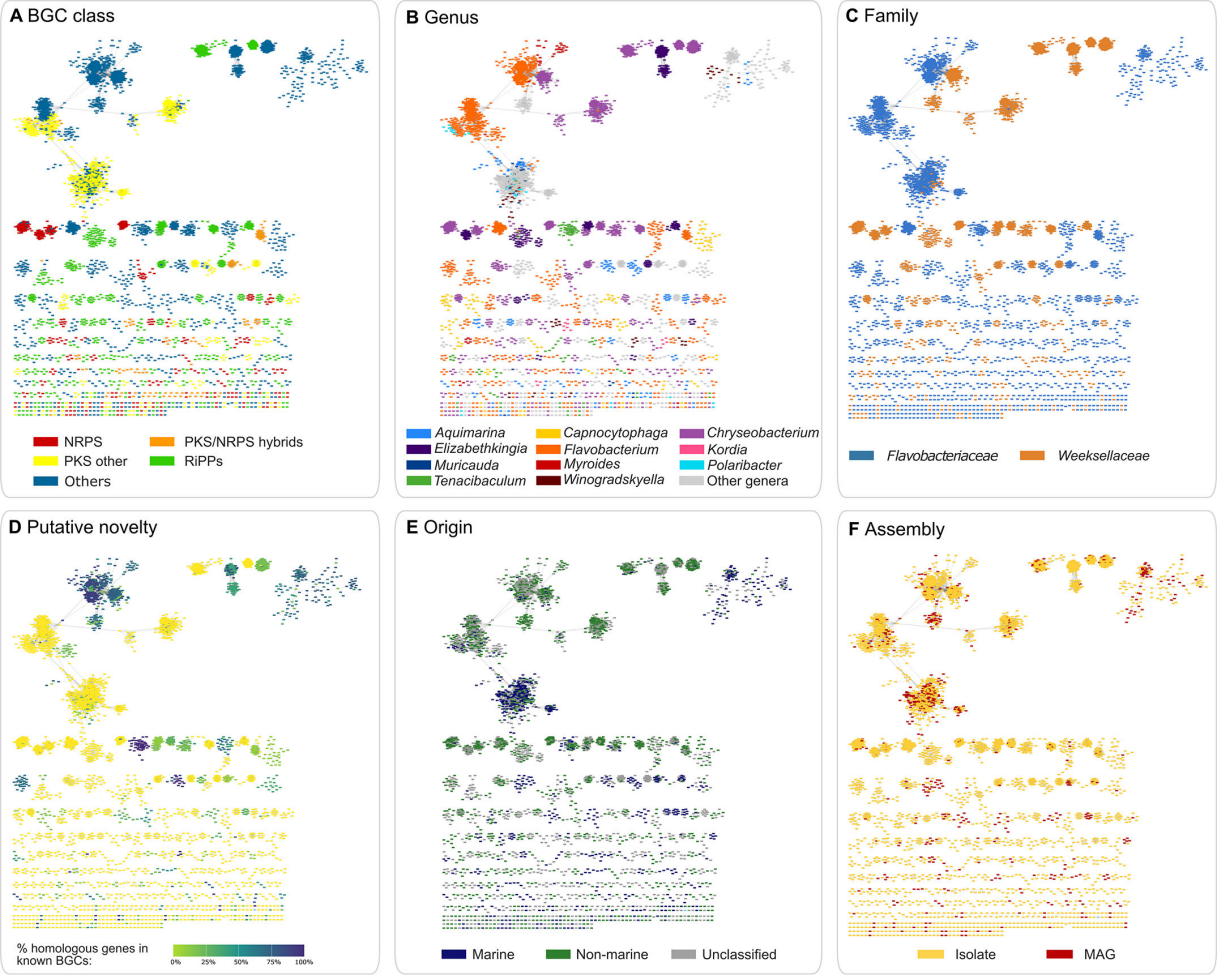
BGC similarity network across *Flavobacteriaceae* and *Weeksellaceae* genomes. Each dot in the network space represents a BGC. BGCs sharing distances below or equal to 0.3 are clustered into GCFs, and GCFs sharing distances between 0.3 and 0.7 are connected in the network space to form GCCs as implemented by the BiG-SCAPE analytical workflow (see Materials and Methods). In each panel, the same network is shown with color legends representing different metadata fields. (A) BiG-SCAPE classification scheme for major compound classes, except terpenes (for terpene-specific similarity networks, please see Fig. S4). (B) Genus-level taxonomy. All genera represented by at least 50 genomes or by a mean number of BGCs per genome higher than seven are colored. (C) Family-level taxonomy. (D) Putative novelty expressed by the proportion of homologous genes found in MIBiG BGCs compared to the BGCs uncovered in this study. (E) Origin. (F) Genome assembly type. Singleton BGCs were excluded from the networks.

**Fig 6:**
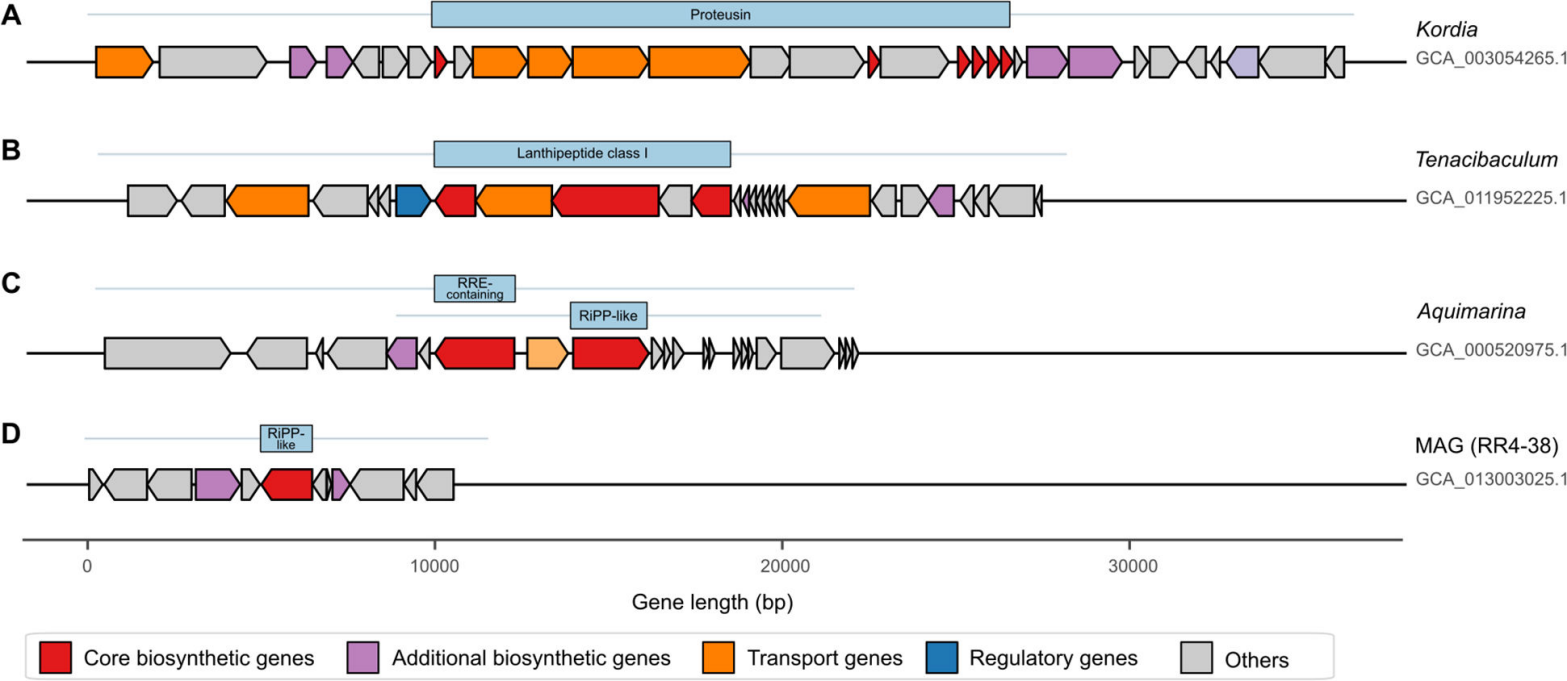
Gene architecture of genus- or assembly type-specific RiPP BGCs. Predicted gene functions are color coded. Gene length, at scale, is represented by the *x*-axis (in base pairs). BGCs are in descending order regarding their full length. Each arrowed rectangle corresponds to a coding sequence (CDS), and the blue bars above represent the respective protoclusters (core biosynthetic genes). The direction of gene arrows represents gene orientation (forward: to the right; reverse: to the left). Gene cluster boundaries (full length represented by thin blue lines) were predicted by antiSMASH using protocluster type-specific cut-offs. Abbreviations: RiPP, ribosomally synthesized and post-translationally modified peptide; RRE, RiPP recognition element.

The identified BGCs were subjected to network analysis using the BiG-SCAPE similarity metric and default clustering parameters, whereby BGCs possessing similarities >70%, the threshold used to classify BGCs in GCFs, are linked. Overall, closely related BGCs formed well-delineated GCFs following the compound classes they putatively encode (Fig. 5A). The juxtaposition of BGCs from the *Flavobacteriaceae* and *Weeksellaceae* families in the same GCF was rarely observed. We rather found that the grouping and distribution of similar BGCs often matched the taxonomy of their bearing genomes at the genus and family levels (Fig. 5B and C). Of the 9,330 BGCs and 2,297 GCFs found in this study, 8,037 BGCs (86.1%) belonged to 2,162 genus-specific GCFs (94.1% of the GCFs in the data set). Among the genus-specific GCFs, 1,255 were singletons (that is, GCFs composed of one single BGC). Of the 102 GCCs identified in the study (none of which were singletons), 78 were found to be genus-specific, and 6,146 BGCs (65.9%) were assigned to such GCCs (Table S6). One-way permutational analysis of variance (one-way PERMANOVA) revealed that GCC and GCF genomic profiles, representing the counts of BGCs assigned to each GCC and GCF on each genome (see Materials and Methods for details), respectively, varied significantly among genomes belonging to different genera (GCC profiles, *P* = 0.001; GCF profiles, *P* = 0.0001). Taxonomy-BGC diversification relationships were observable among terpene-encoding BGCs (Fig. S4A and B) and BGCs involved in RiPP, PKS, and NRPS biosynthesis. For instance, after discarding singleton GCFs, several unique GCFs of closely related RiPP BGCs specific to the genera *Elizabethkingia* (*n* = 14), *Tenacibaculum* (*n* = 6), *Kordia* (*n* = 3), *Aquimarina* (*n* = 7), and *Chryseobacterium* (*n* = 56), among others, were unveiled (Fig. 5A and B; see also Fig. 6). We also observed a few GCFs of NRPS BGCs specific to the genera *Elizabethkingia* (*n* = 2), *Tenacibaculum* (*n* = 4), *Flavobacterium* (*n* = 29), and *Chryseobacterium* (*n* = 19) (singletons excluded; Fig. 5A and B), corroborating findings obtained at the GCC level of resolution (Fig. 4). Unique groups of PKS/NRPS hybrid BGCs were exclusive to genera such as *Kordia*, *Flavobacterium,* and *Winogradskyella* (Fig. 5A and B). A variety of RiPP (*n* = 29), NRPS (*n* = 29), PKS (*n* = 26), and PKS/NRPS hybrid (*n* = 41) GCFs specific to the genus *Flavobacterium*, the most sampled taxon in this study (*n* = 589 genomes), were uncovered (singletons excluded; Fig. 5A and B). The marine genus *Aquimarina*, found in this study to harbor, along with the genus *Kordia*, the highest number of BGCs per genome (Fig. S4), also contained several specific RiPP (*n* = 7), NRPS (*n* = 4), PKS (*n* = 5), and PKS/NRPS hybrid (*n* = 19) GCFs (singletons excluded; Fig. 5A and B). The degree of sharedness of homologous genes between BGCs from this study and MIBiG BGCs was most often below 60% for all compound classes examined (Fig. 5D; Fig. S4C; see also Fig. 3). BGC grouping according to origin was also observed in spite of the lack of provenance metadata for a large proportion (36.94%) of the genomes, with several GCFs composed predominantly of BGCs from either marine or terrestrial settings (Fig. 5E), also in the case of terpene BGCs (Fig. S4D). GCFs composed exclusively of BGCs obtained from MAGs could be observed (Fig. 5F), especially in the case of GCFs involved in the biosynthesis of terpenes (Fig. S4E). These BGCs were, moreover, usually present on MAGs affiliated with so-far uncultivated candidate genera within the *Flavobacteriaceae* family (Fig. S4A, B, and E). We found that the diversity of carotenoid-encoding GCFs was highly congruent with genus-level taxonomy, as many such GCFs were exclusive to genera such as *Flavobacterium*, *Chryseobacterium*, *Elizabethkingia*, *Polaribacter*, *Tenacibaculum*, *Aquimarina,* and *Kordia*, among others (Fig. S4A and F).

To zoom in on the organization and architecture of genus- or assembly type-specific GCFs, representative BGCs from four GCFs of the RiPP class were selected because of their high frequency across the genomes examined and their relevance for natural product research. Three of them were genus specific, GCF 5558 (*Kordia*, two BGCs), GCF 6775 (*Tenacibaculum*, five BGCs), and GCF 10565 (*Aquimarina*, nine BGCs), while one was assembly type specific, GCF 8647 (MAGs only, four BGCs) (Fig. 6). The representative *Kordia*-specific BGC (Fig. 6A) was inferred to encode a proteusin-like compound, a highly modified large leader peptide of the polytheonamide or landornamide type. The representative *Tenacibaculum*-specific BGC was found to encode a class I lanthipeptide (Fig. 6B). Both BGCs representative of the *Aquimarina*-specific (Fig. 6C) and MAG-specific (Fig. 6D) GCFs were identified to contain a “RiPP-like” core of genes (also denoted “protocluster”). However, the first also includes an additional protocluster of the “RiPP recognition element (RRE)-containing” type, being an example of a BGC composed of two neighboring candidate clusters.

### Carbohydrate- and peptide-degrading capacities

The peptide and polysaccharide catalytic potential predicted from the data set was large, as 1,444 peptidase and 749 CAZyme categories were annotated in total (Table S7). Twenty CAZymes and 29 peptidases, representing distinct functionalities and molecular targets, were differentially abundant in marine vs non-marine *Flavobacteriaceae* genomes (*n* = 1,251) following our feature selection pipeline (Fig. S5; Table S8 and S9; File S1). While genes coding for enzymes that participate in the degradation of alginate (PL7_5), rhamnose (GH78), xyloglucan (GH74), fucose (CBM47), and inulin (CBM38) were enriched in marine genomes, in non-marine genomes, genes coding for GT51 and GH25 enzymes that target peptidoglycan, an important component of bacterial cell walls, were more prevalent (Fig. S5A; see File S1 for more details). Several MEROPS features were found to discern between genomes of marine vs non-marine origin, including a higher frequency of collagenase-encoding genes (MER0003242 and MER0013876) among non-marine genomes and carboxypeptidase-encoding genes (MER0002007) among marine genomes (Fig. S5B).

**Fig 7:**
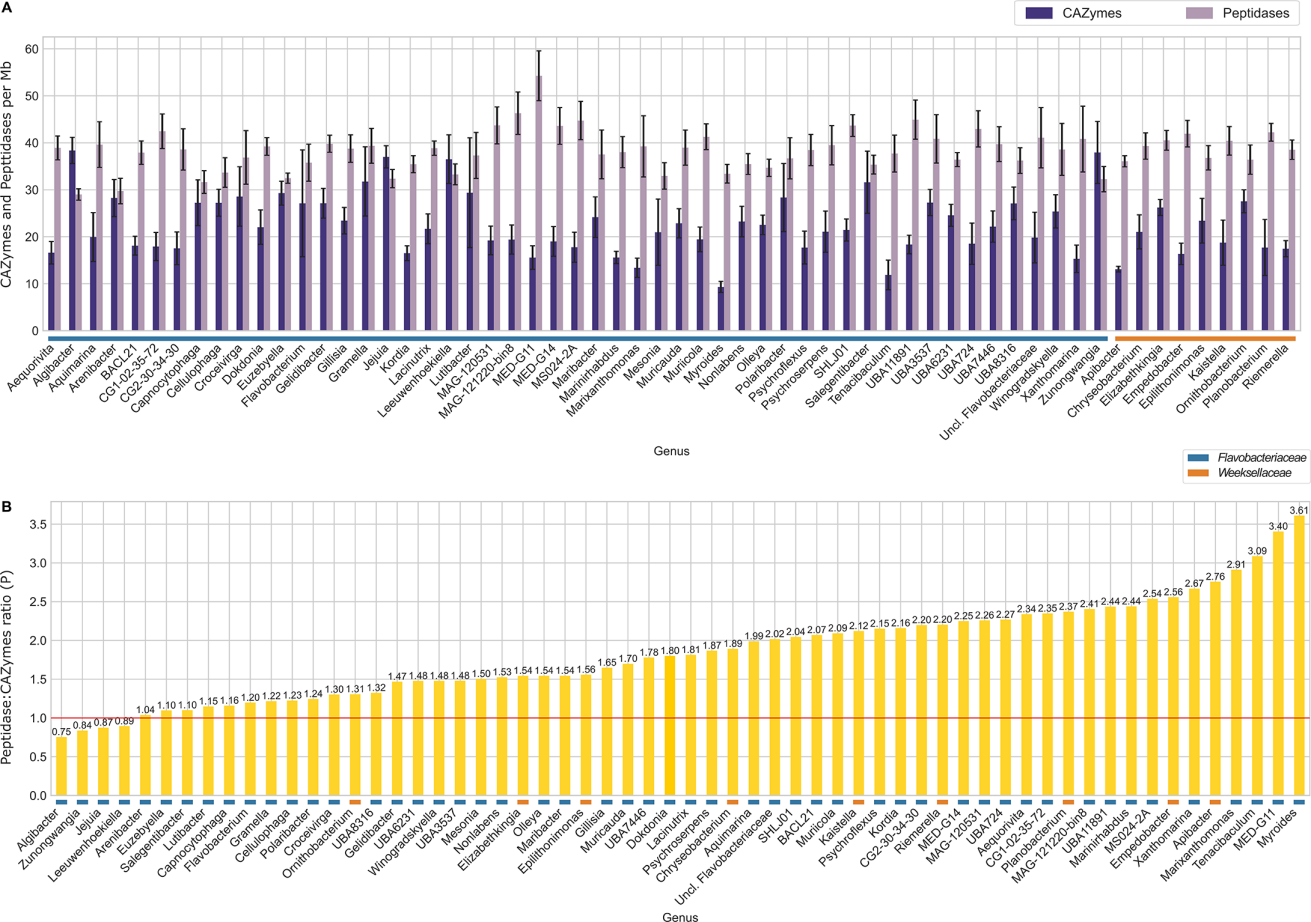
Abundance distribution of CAZymes and peptidases in the data set. Only genera represented by at least eight genomes are presented. (A) Mean CAZyme (dark purple) and peptidase (light purple) counts per genome size (Mb) per genus. (B) Peptidase:CAZyme ratios. Parameters in panels A and B were measured as defined by Fernández-Gómez et al. (4). In panel B, values below 1 (red line) indicate a higher proportion of CAZymes, while values above this number point to a higher proportion of peptidases, indicative of animal host colonization potential or predisposition to oligotrophic habitats, as suggested by Zhang et al. (5). While this taxon-oriented approach revealed genus-specific trends in peptidase:CAZyme proportions across the data, we observed overall no differences when genomes of marine vs non-marine provenance were compared (Fig. S6).

The absolute number of peptidases and CAZymes found per genome was positively correlated with genome size, with a stronger trend for peptidases (Spearman’s correlation I = 0.909) than for CAZymes (Spearman’s correlation I = 0.809) (Fig. S6A and B). The abundance of peptidase- and CAZyme-encoding genes, both per genome and relative to genome size (Mb), varied significantly among bacterial genera (Kruskal-Wallis, *P* < 0.0001), ranging from about 29 to 54 peptidase CDs per Mb in the genera *Algibacter* and MED-G11, respectively, and from nine to 38 CAZyme CDs per Mb in the genera *Myroides* and *Algibacter*, respectively, within the panel of 60 genera containing ≥8 genomes in the study (Fig. 7A). The mean number of peptidases per Mb was higher than that of CAZymes in all analyzed genera except for the genera *Algibacter*, *Jejuia*, *Leeuwenhoekiella*, and *Zunongwangia*, which presented peptidase:CAZyme ratios < 1 (Fig. 7A and B). Peptidase:CAZyme ratios ranged from 0.75 in the genus *Algibacter* to 3.61 in the genus *Myroides* (Fig. 7B), revealing a taxon-dependent variation of this parameter across several dozens of genera within the *Flavobacteriaceae* and *Weeksellaceae* families.

When genomes from different taxa were pooled according to their provenance (i.e., marine, non-marine, or unclassified), we found that a higher number of peptidases and, more pronouncedly, CAZymes were encoded by terrestrial than marine genomes (Fig. S6C; Kruskal-Wallis followed by Dunn’s *post hoc* test, *P* < 0.0001). When the data were normalized by genome size, however, slightly higher frequencies of peptidase-encoding genes were uncovered in marine vs non-marine genomes (Kruskal-Wallis followed by Dunn’s *post hoc* test, *P* < 0.0001), while no significant differences were observed for CAZymes (Kruskal-Wallis followed by Dunn’s *post hoc* test, *P* = 0.9929) (Fig. S6D). Altogether, peptidase:CAZyme ratios did not vary according to organismal origin, that is, marine vs non-marine genomes (Fig. S6E; Kruskal-Wallis followed by Dunn’s *post hoc* test, *P* = 0.1714).

## DISCUSSION

In this study, we perform a comprehensive, large-scale, and taxon-oriented survey of natural product biosynthesis potential within the *Flavobacteriaceae* family, a species- and genus-rich clade of the order *Flavobacteriales* within the *Bacteroidota* phylum (15). Recent comparative genomic studies of *Flavobacteriaceae* species have been instrumental in improving our understanding of carbohydrate and peptide catabolism as adaptive traits, leading to niche partitioning and diversification within this group (16, 17). These surveys have been applied to a comparatively smaller number of genomes covering a limited spectrum of the large diversity within the family. Also, the coding potential of uncultivated *Flavobacteriaceae* lineages (MAGs) has not been contemplated in earlier functional genomic studies of *Flavobacteriaceae* or *Bacteroidota* species at large. Here, we overcome previous limitations of genome-wide surveys of the *Flavobacteriaceae* family by performing a large-scale analysis of 2,680 genomes, including genomes from uncultured organisms in the form of MAGs (25% of the genomes in this data set). We found that 38.73% of the MAGs examined here were not assigned to any formally described genera, while those assigned to formally described genera sharply increased the known diversity of cultivatable or “hard-to-culture” and poorly understood groups. Although the absolute number of BGCs found on MAGs was overall lower than on isolate genomes, probably because MAGs are usually assembled with higher numbers of shorter contigs, we found no differences in BGC counts per Mb among these groups. Innovative culturomics and heterologous BGC expression approaches hold promise in improving natural product discoverability among so-far uncultivated lineages of *Flavobacteriaceae*.

### A large diversity of BGCs encode putatively novel carotenoid and flexirubin pigments

Pigments represent a large portion of the end-products of the identified BGCs. Carotenoids are a diversified group of naturally occurring terpenoid pigments that are among the most widespread natural products (18). These compounds, exclusively produced by plants and microorganisms, play important biological roles as accessory light-harvesting components in photosynthetic systems, as photo-protecting antioxidants, regulators of membrane fluidity, and in vitamin A biosynthesis. There are at least 300 known carotenoids (18), and this diversity arises from countless possible combinations of oxidation, oxygenation, cyclization, and glycosylation steps in their biosynthetic pathways. During carotenoid evolution, structural diversification seemed to have emerged from later biosynthetic steps compared to those closer to the biosynthetic ‘‘root,’’ while core carotenoid biosynthetic genes are usually highly conserved functionally and phylogenetically (19). Our findings align with this perspective and the potential proneness for diversification among carotenoid pigments, as also suggested by a recent, large-scale study of the biosynthetic potential of the ocean microbiome (20). Here, we place this perspective for the first time into a well-delineated, taxon-oriented data analysis framework to uncover an unprecedented diversity of carotenoid-encoding BGCs. These BCGs encompass multiple, genus-specific gene cluster clans and families, including several GCFs exclusively found in so-far uncultured taxa represented by MAGs. The extent to which the here depicted heterogeneity in carotenoid-encoding BGCs translates into diverse carotenoid molecular structures and, by extension, the differential adaptability of carotenoid-producing taxa to the environment is yet to be elucidated. Our outcomes open new and exciting avenues to studying the roles and diversification of carotenoids as fitness-enhancing factors in bacteria, suggesting that a larger repertoire of naturally occurring carotenoids may be available for biotechnological exploitation than previously thought.

The second most abundant compound encoded by BGCs from our data set is the pigment flexirubin, which is only found in the *Bacteroidota* phylum, oftentimes used as a chemotaxonomic marker (1). However, here, we show that not all members of the *Flavobacteriaceae* and *Weeksellaceae* families possess flexirubin-encoding BGCs. We found several genus-specific (e.g., *Flavobacterium*, *Chryseobacterium*, *Elizabethkingia*, and *Empedobacter*) GCCs putatively involved in flexirubin biosynthesis, hinting at phylogeny-driven diversification of flexirubin BGCs within the *Bacteroidota* phylum in a similar fashion as discussed above for carotenoids. The pigment flexirubin is an aryl polyene of the dialkylresorcinol type. It is structurally similar to carotenoids with respect to their polyene system and also protects cells from oxidative stress (21).

The widespread capability of synthesizing pigments conveying protective functions, such as shielding from irradiation, and mechanisms to cope with oxidative and osmotic stress confers bacterial cells with survival advantages in diverse environments. This study uncovers compelling evidence of taxon-driven structural heterogeneity of carotenoid- and flexirubin-encoding BGCs, suggesting that the full molecular diversity within both pigment types is far from being exhausted. Our findings also indicate that both traits are fundamental pillars reflecting phylogenetic diversification and adaptive evolution in the *Flavobacteriaceae* family beyond peptide and carbohydrate degradation versatility.

### *Flavobacteriaceae* BGCs code for compounds with diverse eco-physiological functions and pharmaceutical properties

The *Flavobacteriaceae* genera *Aquimarina*, *Flavobacterium,* and *Tenacibaculum* and the *Weeksellaceae* genera *Chryseobacterium* and *Elizabethkingia* were rich in BGCs coding for siderophores such as bisucaberin B, desferrioxamine, and putrebactin/avaroferrin-related compounds. Siderophores are usually low-molecular-weight compounds secreted by the cell to the extracellular environment in response to iron limitation to sequester and solubilize iron from the environment or the host (22). Desferrioxamine E (DFO-E, also nocardamine) was first isolated in 1982 from *Streptomyces* spp. (23) and subsequently found in many other bacteria. Bisucaberin B, first discovered in *Alteromonas haloplanktis* isolated from deep-sea mud (24), is known to halt tumor growth by withholding cellular iron and promoting macrophage-mediated cell cytolysis. Siderophores are important virulence factors in many pathogenic bacteria by altering iron homeostasis, depriving the host cell of vital iron and other micronutrients, and even modulating host cellular pathways (25). This finding indicates how genera such as *Aquimarina*, *Tenacibaculum,* and *Chryseobacterium* may be successful opportunistic pathogens. In addition, siderophore secretion in densely populated microbiomes and niches, such as marine biofilms or algal blooms, may mediate inter-species competition and provide siderophore-producing *Flavobacteriia* with a competitive advantage by depriving other bacteria of this essential element (26).

Several bioactive compounds were inferred to be encoded by BGCs detected in this study, showing high homology with experimentally validated BGCs. Some of these compounds have not yet been (to the best of our knowledge) reported to be produced by species of the *Flavobacteriaceae* and *Weeksellaceae* families or of the *Bacteroidota* phylum altogether. These include the antibacterial compounds rhizomide, a cyclic lipopeptide (NRPS) originally isolated from *Paraburkholderia rhizoxinica* HKI 454 (*Betaproteobacteria*, *Burkholderiaceae*) (27), and xenematide, a cyclodepsipeptide (NRPS) first described from the nematode-associated entomopathogenic bacterium *Xenorhabdus nematophilus* (*Gammaproteobacteria*, *Morganellaceae*) (28). Also, the BGC coding for the protease inhibitor anabaenopeptin NZ857, a non-ribosomal peptide known to be produced by *Nostoc punctiforme* (*Cyanobacteria*), was recently reported for the first time in the *Aquimarina* (*Flavobacteriaceae*) genome (29). The abovementioned BGCs were, in this study, detected in the genomes of several *Flavobacteriaceae* genera (see Results for details). Altogether, these data suggest that horizontal gene transfer events may mediate the transit of some BGCs across *Flavobacteriaceae* genera and beyond, independent of the strong phylogenetic signal underpinning BGC richness and genus-level taxonomy within this family. It is important to note that the genus-specific occurrence of BGCs, GCFs, and GCCs, as reported in this study, can be influenced by “sampling effort,” as some genera were better represented with a large number of genomes than others, and exhaustive BGC documentation may have been hampered among poorly represented genera.

Compounds from large and structurally diverse families of secondary metabolites, such as polyketides, non-ribosomal peptides, and post-translationally modified peptides (RiPPs), are of interest both for their diverse ecological functions and their eventual use as sources of novel drug-like compounds. It is hypothesized that, especially in high cellular density environments, the biosynthesis of inhibitory molecules may play a role in competitive and anti-predatory interactions in nature, constituting an asset for symbiotic microorganisms, be it in a pathogenic or rather mutualistic fashion. For instance, microbial symbionts may aid eukaryotic hosts with chemical defense against natural enemies. One prominent example of such a mutualistic relationship is the biosynthesis of anti-predatory bryostatin polyketides by a gammaproteobacterial symbiont of the bryozoan *Bugula neritina* (30). On another note, a higher prevalence of homoserine lactone-, NRPS-, RiPP-, and siderophore-encoding BGCs has been reported for diseased compared to healthy coral tissue and suggested to result from intense competition between opportunistic bacteria during the process of dysbiosis and secondary colonization of the decaying coral host (31).

Following BGCs coding for carotenoid and flexirubin pigments, RiPP-encoding BGCs constituted the most abundant BGC type across the data set. In this study, BGCs coding for lanthipeptides were used to showcase, in greater detail, the general correspondence between phylogeny and secondary metabolism unveiled across the data (Fig. 6). Lanthipeptides, which are characterized by the presence of lanthionine (Lan) and methyllanthionine (MeLan) residues, are one of the most studied types of RiPPs (32). A plethora of activities has been assigned to these compounds, including antifungal, antiviral, and antiallodynic activities, besides the prominent antimicrobial action that characterizes some compounds of this group, historically called lantibiotics (32). Figure 6 presents a *Tenacibaculum*-specific BGC lanthipeptide as a potential target for future drug discovery efforts within the lantibiotics class. It also presents a proteusin BGC from *Kordia*. Proteusins are a family of RiPPs whose small BGCs are often overlooked in genome mining efforts. Moreover, these clusters are often silent under axenic laboratory conditions, which further contributes to the lack of knowledge of this compound family. Important members of the proteusin family are the “*Candidatus Entotheonella*”-derived polytheonamides and the antiviral landornamides (33).

### The marine genera *Aquimarina* and *Kordia* stand out as prolific sources of secondary metabolites within the *Flavobacteriaceae* family

The potential for secondary metabolite production is not evenly spread across the numerous genera examined here. Instead, three genera stood out in this study regarding secondary metabolism versatility: the marine genera *Aquimarina* and *Kordia* (*Flavobacteriaceae*) and the *Weeksellaceae* genus *Chryseobacterium*. Our earlier comparative genomic study of species in the *Aquimarina* genus revealed an unexpected, diverse secondary metabolism for this taxon (6). We observed that BGC profiles mirrored phylogenomic and functional genomic relationships across *Aquimarina* species, suggesting correspondence between natural product biosynthesis potential and microevolutionary trajectories (i.e., species-level diversification and evolution) within a narrow taxonomic framework. The present large-scale analysis of thousands of genomes encompassing 175 genera within the order *Flavobacteriales* now strengthens the notion that secondary metabolism traits may as well reflect macroevolutionary trajectories within large organismal clades, as GCF and GCC abundance distributions were found to change significantly across *Flavobacteriaceae* genera. In fact, beyond the carotenoid and flexirubin GCFs mentioned above, multiple GCFs involved in the biosynthesis of several compound classes, including those used as sources of new scaffolds for drug development inspired by nature, such as NRPs, RiPPs, and polyketides, were found to display taxon-specific structural diversity.

This study provides additional evidence about the unique biosynthetic potential of the genus *Aquimarina*, revealing it as one of the most prolific secondary metabolite producers in the *Flavobacteriaceae* family. The observation that species within the genus *Kordia,* the closest relative to *Aquimarina,* have as well displayed highly complex BGC profiles reinforces the notion that phylogenetic relationships within the family underpin secondary metabolism profuseness. The recent discoveries of the polyketide cuniculene and the antibiotic peptides aquimarins from *Aquimarina* species exemplify how genome mining for BGCs may aid in the structural elucidation of novel molecular scaffolds (34, 35). According to the *in silico* predictions made by the reports of Silva et al. (6, 29) and in this study, several novel compounds from diverse classes are yet to be unveiled from this genus. Members of the genus *Kordia*, in their turn, play a vital role in the marine carbon cycle since they feed on phytoplankton particles and possess algicidal activities, contributing to the control of phytoplankton blooms (36). Despite their relevant roles in nature, knowledge of secondary metabolites typically produced by *Kordia* species is acutely scarce, if non-existent. Altogether, our study reveals pronounced biosynthetic potential for some readily culturable bacterial taxa through the analysis of thousands of genomes from the *Flavobacteriaceae* family, including genera such as *Croceivirga*, *Dokdonia*, *Euzebyella*, *Flavobacterium*, *Maribacter,* and *Tenacibaculum,* which, after *Aquimarina* and *Kordia*, also presented diverse BGC profiles. The paucity of validated secondary metabolites reported from these genera highlights the potential of prioritizing natural product research on less explored bacterial taxa to respond swiftly to timely public health demands such as the antimicrobial resistance crisis.

When placed into a broader context, the most promising *Flavobacteriaceae* genera possess a rather moderate biosynthetic potential when compared to well-established, highly prolific bacterial taxa such as the genus *Streptomyces* (phylum *Actinomycetota*) and the order *Myxococcales* (phylum *Myxococcota*). Strains in these groups may present up to 83 (37) and 52 (38) BGCs per genome, respectively, according to recent genome mining estimates. Larger genomes also tend to possess higher BGC counts within these taxa, whose genome sizes can reach up to 13–14 Mb for some strains (38), roughly two times larger than the largest *Flavobacteriaceae* genomes identified in our data set.

### Marine and non-marine *Flavobacteriaceae* genomes are differentially enriched in CAZymes and peptidases

We used a machine learning pipeline to identify the polymer-degrading enzymes that are indicators of adaptation to either marine or non-marine environments among *Flavobacteriaceae* species. In the marine group, genomes were enriched in genes of the enzyme families xyloglucanases (GH74) and rhamnosidases (GH78). Xyloglucans are hemicellulose polysaccharides not only present in the cell wall of all major groups of land plants but also present in charophycean green algae, while rhamnose is a glycoprotein produced by bacteria, plants, and algae. Moreover, alginate lyases (PL7_5), fucolectins, and fucose-binding proteins (CMB47) were enriched in marine *Flavobacteriaceae*. Alginate is an abundant heteropolysaccharide of the cell walls and intracellular material of brown algae, while fucoidans are a complex series of sulfated polysaccharides, also abundant in brown algae. Sulfated polysaccharides are known to be absent in terrestrial plants but common in marine ecosystems. These negatively charged molecules, which are common in seaweeds, possess structural stability even under higher-salinity conditions. The non-marine *Flavobacteriaceae* genomes were enriched in enzymes of the family GH19, known for its endo-chitinase and lysozyme activity, and GH25, a group of peptidoglycan-degrading lysozymes involved in cell wall remodeling. Both GH families are known to be involved in host cell wall invasion by pathogenic bacteria (39, 40). A detailed discussion on the relevance of this parameter to infer opportunistic behavior and its relationship with genome size and evolution is provided in File S1.

### Catabolic and secondary metabolite potential, along with genome size, are likely intertwined features dictating niche differentiation within the *Flavobacteriaceae* family

We also uncovered significant shifts in the relative abundance of peptidase- and CAZyme-encoding genes across multiple *Flavobacteriaceae* genera, revealing taxon-dependent catalytic profiles suggestive of the adaptive evolution of these organisms to distinct microniches. Indeed, peptidase:CAZyme ratios have been suggested as proxies for habitat conditions and microbial lifestyles (41). High peptidase:CAZyme ratios seem to be more prevalent among bacteria often associated with hosts or isolated from oligotrophic habitats (5). This suggests that opportunistic bacteria living in the open environment may be capable of invading hosts under favorable conditions, establishing a biphasic lifestyle relying on their aptitude to thrive in open and host-associated habitats. Here, we observed that the genera *Aquimarina* and *Kordia* possessed versatile peptide degradation potential and larger genomes than other *Flavobacteriaceae* genera. These taxa, besides possessing the extensive carbon-degrading capabilities commonly observed among *Flavobacteriaceae* species, have a repertoire of peptidases that likely allows them to also persist in oligotrophic environments, such as open seawater, where both carbon and nitrogen availability are commonly reduced. The combination of a diversified secondary metabolism with versatile carbohydrate and peptide degradation capacities presumably makes these bacteria formidable host colonizers that can engage in opportunistic-to-pathogenic behavior while proliferating in these niches. For instance, growth on chitin—a major biopolymer present in fungal cell walls and as a structural component of crustaceans, insects, and phytoplankton—was found to induce secondary metabolite gene expression in the soil-dwelling actinomycete *Streptomyces coelicolor* (42) and among *Vibrionaceae* spp. (43), where it promoted the biosynthesis of the antibiotic andrimid by the coral pathogen *Vibrio coralliilyticus* (44). Unveiling the extent to which secondary metabolite biosynthesis and catabolic traits are interlinked among *Flavobacteriaceae* species holds promise in illuminating the coregulatory networks underlying their ecophysiology and adaptive metabolism.

### Conclusions

Recent advances in genome biology, including the development of genome mining algorithms for the detection of BGCs, have spurred the rate of discovery of microbially produced natural products, but the extent to which their diversity recapitulates macroevolutionary trajectories within broad organismal clades has been seldom examined. We reveal unprecedented taxon-dependent diversification of BGCs in the families *Flavobacteriaceae* and *Weeksellaceae*, key drivers of biogeochemical cycles worldwide, across broad biosynthetic scenarios. Indeed, clustering of BGCs involved in the biosynthesis of terpenes (including carotenoids), flexirubins, polyketides, non-ribosomal peptides, and ribosomally synthesized and post-translationally modified peptides was largely family- and/or genus-specific. BGC profiling across MAGs revealed a wealth of carotenoid-encoding biosynthetic gene clusters unique to several uncultivated clades representing novel candidate lineages within the *Flavobacteriaceae* family. Moreover, our approach breaks new ground in recognition of some *Flavobacteriaceae* taxa, otherwise mostly known for their versatile catabolic traits, as promising renewable sources of novel drug leads. This may be particularly valid for genera such as *Aquimarina* and *Kordia* that show a remarkable potential to synthesize diverse secondary metabolites and thus shall be targeted more thoroughly in future bioprospecting campaigns for novel microbially synthesized drugs.

## MATERIALS AND METHODS

### Data set creation and genome selection

Genome assemblies (contig files) affiliated with the class *Flavobacteriia* (taxid = 117,743, *n* = 3,493 genomes, stand: September 2020) were downloaded in fasta format from GenBank-National Center for Biotechnology Information (NCBI). GTDB-Tk v.1.3.0 (45) was used to perform a fresh taxonomy assignment of all genomes obtained, and those not classified into the *Flavobacteriaceae* or *Weeksellaceae* families (*n* = 667) were discarded, resulting in 2,826 genomes. No genome dereplication was conducted in this study to maximize BGC discoverability, as highly similar genomes/MAGs from independent studies may share different sets of auxiliary genes that might get lost upon dereplication (46). The primarily terrestrial *Weeksellaceae* family, the closest taxonomic relative to the *Flavobacteriaceae* family, was included in this study to serve as a phylogenomic outgroup and enable a more extensive mining of BGCs among genomes of terrestrial and marine origin within the *Flavobacteriales* order. The genomes were further subjected to quality control and filtering, and genome completeness, contamination, and strain heterogeneity were obtained using CheckM v1.0.7 (47). A quality score was calculated for each genome according to Parks et al. (48), where quality = completeness (%) − 5 × contamination (%) . The statswrapper tool from BBTools suite v38.00 (sourceforge.net/projects/bbmap/) was employed to acquire additional genome metrics (e.g., number of contigs, average contig length, GC content, and N50, among others; Table S1), which were also used to classify the genomes regarding quality. First, all genomes with an overall quality score ≤50%, completeness score ≤50%, and/or contamination ≥10% were considered low quality (*n* = 146) and discarded from further analysis. We then grouped the remaining genomes into excellent-, high-, and medium-quality categories. “Excellent-quality” genomes possessed completeness ≥90%, contamination ≤5%, strain heterogeneity ≤10%, and ≤50 contigs. If completeness was between 80% and 90%, contamination between 5% and 10%, and strain heterogeneity ≤50%, genomes were classified as “high quality.” “Medium-quality” genomes all possessed more than 50% completeness, less than 10% contamination, and an overall quality score of >50%, thus complying with current stringency standards for comparative genomic studies (49). The final data set was composed of 2,680 genomes from medium to excellent quality, as determined above.

### Metadata mining and standardization

To retrieve extensive metadata for all genomes, four data sources were exploited: GenBank, PATRIC (50), BioSamples (51), and Sequence Read Archive (SRA) (52). First, metadata available upon downloading genomes from NCBI/GenBank were compiled. Then, all available metadata for genomes of the class *Flavobacteriia* included in the PATRIC database were downloaded from the PATRIC website (https://patricbrc.org/). To collect information contained in the BioSamples database, publicly available at https://www.ebi.ac.uk/biosamples/, a manual search for all genomes of the *Flavobacteriales* order was performed. Then, an XML file including all available attributes was extracted and converted into a readable format using the script accessible at https://github.com/transcript/BettaMetaData/tree/master/data/ncbi-biosample. Finally, to recover metadata from SRA, the SRAdb v1.48.2 R package (53) was used to access metadata entries from SRA locally. The metadata collected at all these steps were merged and curated with a custom-made Python pipeline, available at https://github.com/sandragodinhosilva/flavobacteriaceae_project. To minimize the occurrence of missing values, we discarded all metadata attributes present in less than 10 genomes from the data set. The final metadata table was composed of 162 attributes (Table S2). Thereafter, we designed a workflow to fill an extra metadata attribute in the table, denoted “origin,” to record the provenance of genomes according to terms provided by submitters that matched custom dictionaries created, in this study, for marine, non-marine, and transition environments based on the Environment Ontology (ENVO) (54). In addition, manual exploration for metadata keywords (Table S3) was employed to record the metadata field “origin.” When available, geographical coordinates provided by submitters were used to differentiate between terrestrial and marine sampling sites with the aid of the Python package global-land-mask v0.0.3 (https://pypi.org/project/global-land-mask/). To prevent misclassification of genomes regarding their origin, terms related to transition environments, such as “tidal flats” and “hypersaline environments,” were not used to ascertain genome origin as marine or terrestrial.

### Whole genome sequence clustering

A whole genome-based cladogram was constructed using Mashtree v1.1.2 (13) to display the relatedness and diversity of bacteria classified into 60 genera (from both the *Flavobacteriaceae* and *Weeksellaceae* families) found to be represented by ≥8 genomes in the data set. Collectively, these genera encompassed 2,359 (88%) of the 2,680 genomes annotated in this study and were chosen as a representative group to display major taxon-dependent genomic features across the data in a tractable fashion (Fig. 1, 2, 3, and 7), whereas statistical handling of the data encompassed all genera and genomes in the study (see details below). They have also been used as input data to determine whether shifts in the relative abundances of peptidase- and CAZyme-encoding genes per genus were significant (Fig. 7A; see details below). Briefly, Mashtree computes a pairwise distance matrix for any group of genomes based on sketches composed of hashed k-mers, created and sorted for each genome with the MinHash algorithm (13, 55). Jaccard distances are then calculated for each pair of genome sketches based on the proportion of hashed k-mers they have in common, and a dendrogram is built thereafter using the neighbor-joining method with the QuickTree tool (56). Clade robustness was tested using a bootstrapping resampling method (100 repetitions) implemented in Mashtree v1.1.2. Visualization of the resulting cladogram was performed in iTOL v4 (57).

### Annotation of biosynthetic gene clusters, CAZymes, and peptidases

The identification of BGCs across all genomes was performed with the standalone version of antiSMASH v5.0 (58) using gbk files as input data. Then, BGC similarity networks were constructed with BiG-SCAPE v1.0.0 (59) using default parameters and visualized in Cytoscape v3.8.1 (60) with the “Perfuse Force Directed Layout.” Briefly, BiG-SCAPE integrates three dissimilarity metrics based on (i) the percentage of shared types of Pfam domains between BGCs; (ii) the degree of synteny between BGCs, measured by the percentage of pairs of adjacent Pfam domains; and (iii) the sequence similarity between the domains identified in different BGCs to compute a global dissimilarity index between pairs of BGCs (59). BiG-SCAPE uses this index to construct networks of BGCs with pairwise distances lower than or equal to a 0.3 distance threshold, forming GCFs. A second clustering layer is performed afterward, grouping GCFs connected by a distance lower than or equal to 0.7 into GCCs.

Metadata information was layered over two major BGC networks constructed in this study dedicated to the analysis of (i) all major BGC classes except for terpene BGCs (Fig. 5A through F) and (ii) terpene BGCs only, owing to their large representation in the data set (Fig. S4A through F). The network analyses were employed to assess the extent to which finely resolved BGC structural diversity corresponds to (i) the compound classes they encode, (ii) taxonomy at the genus and family levels, (iii) putative BGC novelty, (iv) organismal/BGC provenance, and (v) assembly type (isolate genomes vs MAGs). The degree of novelty of the identified BGCs was assessed through matches with BGCs present in the MIBiG database, which lists BGCs leading to the biosynthesis of known compounds with elucidated structures (14). In this study, we use a conservative approach to consider any “likely novel” BGC that shared homologous genes with ≤60% of the genes from validated BGCs present in the MIBiG database. Additional information on the features of known compounds likely synthesized by specific BGCs was obtained from The Natural Products Atlas (61). The networks are available for interactive mining at https://www.ndexbio.org/ using digital object identifiers disclosed in the Data Availability section below.

Genus- or assembly type-specific BGCs encoding likely novel metabolites within a major compound class were selected for further analysis to showcase their synteny and gene architecture, as displayed in Fig. 6. Selection priority was given for BGCs not starting on a contig edge (start base pair different from 1), coming from a type strain and larger size. To analyze selected BGCs, first, antiSMASH region GenBank files were converted into general feature format (GFF) files using Artemis v18.2.0 (62) and then transformed into comma-separated files (.csv) using Python package gffpandas v1.2.0 (https://gffpandas.readthedocs.io/, accessed on 15 July 2022). Gene cluster organization was visualized using R packages gggenes v0.4.1 (https://github.com/wilkox/gggenes/, accessed on 15 July 2022) and ggplot2 v3.3.6 (https://ggplot2.tidyverse.org/, accessed on 15 July 2022).

Besides mining for BGCs, we performed peptidase and CAZyme annotations to comprehensively address the adaptive features of *Flavobacteriaceae* spp. to marine and terrestrial biomes and inspect whether counts of peptidase- and CAZyme-encoding genes shift with genus-level taxonomy. All genomes (contig fasta files) were first annotated with Prokka v1.14.6 (63) for ORF prediction and translation of coding gene sequences into amino acid sequences, after which GenBank (gbk) format and amino acid sequence fasta (faa) format files were obtained. Amino acid sequence (fasta) files were used as input for peptidase and CAZyme annotations across all genomes. The ORFs encoding peptidases were identified with BLASTP (*E*-value 1e−5) against the MEROPS database (64). For CAZyme annotation, a standalone version of the dbCAN2 meta server (65), the run_dbcan v2.0.11 script, was implemented (https://github.com/linnabrown/run_dbcan). This tool runs a HMMER v3.3 search against the dbCAN HMM database (65, 66), a DIAMOND v0.9.32 search against the CAZy database (67, 68), and a Hotpep v2.0.8 search against the conserved CAZyme short peptide database (PPR: https://sourceforge.net/projects/peptide-pattern-recognition/) (69). As suggested by the authors (65), only ORFs for which at least two of the database searches were positive were annotated with the respective CAZyme name. Table S7 lists the number of ORFs, CAZymes, and peptidases predicted for each genome analyzed in this study. CAZyme and peptidase annotation tables were then used to determine the abundance distributions of genes coding for carbohydrate- and peptide-degrading enzymes and estimate CAZyme/peptidase ratios per genome across all genera represented by at least eight genomes in this study.

### Using machine learning to assess catalytic potential as an adaptive feature within the *Flavobacteriaceae* family

A machine learning pipeline composed mainly of FS steps was employed on CAZyme and peptidase annotation tables to identify the most important features distinguishing *Flavobacteriaceae* genomes based on their provenance (marine vs non-marine) (Fig. S7). Briefly, FS can help identify differentiating functions in large-scale genomic data sets reliably, as it reduces data set dimensionality by selecting the features that provide a better separation of two or more target classes (sample groups). While contributing to improving the overall accuracy of the machine learning algorithm, feature selection also removes irrelevant attributes, allowing feature-based discrimination of large groups of genomes (70). To achieve this, we used a random forest (RF) classifier, which is known for its high accuracy, robustness, and ability to handle high-dimensional data, and the performance of the classifier was evaluated using the *F*-measure, which is a commonly used metric to balance precision and recall in classification tasks (71). We used a Python wrapper for the machine learning software Weka (https://pypi.org/project/python-weka-wrapper3/) alongside Google Colab due to the computational power it offers. Feature selection was performed with two distinct data representation modes (counts and presence/absence) for all genomes within the *Flavobacteriaceae* family that were classified as either marine or non-marine. Under the “counts” mode, the total number of each CAZyme and peptidase category annotated per genome is taken as input data in the feature selection procedure, whereas the presence/absence mode registers whether a given feature was annotated per genome. For the feature selection workflow, each data set was divided into training and testing data sets in an 80:20 proportion, and the training and evaluation of the model were performed with an RF classifier. Two sequential steps were performed: (i) correlation-based feature subset selection and (ii) information gain algorithm. The correlation-based feature subset selection considers the individual predictive ability of each feature and subset features that are very correlated with the class (i.e., marine, non-marine, and unclassified) but have a low degree of redundancy between them. In contrast, the information gain algorithm measures the amount of information provided by each attribute (i.e., reduces entropy) for the class examined using the following formula: InfoGain(Class,Attribute) = *H*(Class) − *H*(Class | Attribute), where *H* is a measure of entropy. For feature selection by this method, a set of parameters was tested by the classifier with cross-validation, and the pairs of values that led to a higher *F*-measure value were used for the feature selection by the information gain algorithm. The final output of this pipeline includes the features that, when used together, lead to the best *F*-measure values and are, therefore, considered the essential features to distinguish between classes (that is, groups of genomes in each class).

### Data visualization and statistical analyses

Data analysis and visualization were performed in Python 3 v7.4 using the base packages pandas v1.0.4 and numpy v1.16.6 for data wrangling and the packages seaborn 0.9.0 and matplotlib v3.0.3 for data visualization. The R packages ComplexHeatmap v2.6.0 (72) and ggplot v2 3.1.0 were used to produce a heatmap for the feature selection results in an environment with R v3.5.1. Statistical comparisons were performed in Past v4.06b (73).

Multivariate statistical analyses comprised one-way PERMANOVA to test whether abundance distributions of GCCs and GCFs varied significantly among bacterial genera. To this end, two BGC vs genome contingency tables were produced, each containing the number of BGCs assigned to the (i) GCCs and (ii) GCFs found in this study, respectively, on each genome. The resulting GCC and GCF profiles were then Hellinger transformed (square root of the relative abundance of each GCF and GCC per genome) and used as input data for the calculation of Bray-Curtis similarities between genomes, which were grouped according to their genus-level taxonomy. PERMANOVA was then employed on the retrieved Bray-Curtis similarity matrices to test whether GCC and GCF profiles varied significantly among genome groups (see Data Availability for access to the GCC and GCF profiling tables and the corresponding PERMANOVA results).

For univariate statistical analyses, normality (Shapiro-Wilk test) and equal variance (equal means, Levene’s tests) were first verified across sample groups to allow for adequate test choice following data distribution features. Due to non-normal distributions and unequal variances, possible differences in genome size and in BGC counts (normalized by genome size) between MAGs and isolate genomes were tested with the Mann-Whitney *U* test. To test for differences in BGC counts between genera and in the number of peptidases or CAZymes (normalized by genome size) between genera and across *Flavobacteriaceae* genomes of different provenance (marine, non-marine, and “unclassified”), the Kruskal-Wallis test was employed, followed by Dunn’s *post hoc* test for pairwise comparisons.

## Data Availability

All scripts and codes produced for this study, including data set creation, data analysis, and figure creation, are available at https://github.com/sandragodinhosilva/flavobacteriaceae_project. The GCF and GCC genome profiling tables used as input for multivariate analyses to test whether secondary metabolite biosynthetic potential shifts significantly across *Flavobacteriaceae* and *Weekselaceae* genera and the corresponding PERMANOVA results are available on this same webpage. Figure 5 possesses an online interactive version available in the following links: https://doi.org/10.18119/N9Z60J (Fig. 5A), https://doi.org/10.18119/N9NS44 (Fig. 5B), https://doi.org/10.18119/N9D89P (Fig. 5C), https://doi.org/10.18119/N94S5V (Fig. 5D), https://doi.org/10.18119/N9J02H (Fig. 5E), and https://doi.org/10.18119/N98G7R (Fig. 5F).­­

## SUPPLEMENTAL MATERIAL

File S1 msystems.00643-23-s0001.pdf Supplemental results and discussion.

Fig. S1 msystems.00643-23-s0002.png Data set overview.

Fig. S2 msystems.00643-23-s0003.png Genus-level classification of isolate genomes and MAGs.

Fig. S3 msystems.00643-23-s0004.png Mean BGC counts per genome per genus.

Fig. S4 msystems.00643-23-s0005.png Similarity network of terpene-encoding BGCs.

Fig. S5 msystems.00643-23-s0006.png Distinctive carbohydrate- and peptidase-degrading features among marine and non-marine *Flavobacteriaceae*.

Fig. S6 msystems.00643-23-s0007.png Peptidase and CAZyme distribution per genome size and origin.

Fig. S7 msystems.00643-23-s0008.png Feature selection workflow.

Legends to Supplemental Items msystems.00643-23-s0009.pdf Legends to Fig. S1 to S7 and Tables S1 to S9.

Supplemental Tables msystems.00643-23-s0010.xlsx Tables S1 to S9.

ASM does not own the copyrights to Supplemental Material that may be linked to, or accessed through, an article. The authors have granted ASM a non-exclusive, world-wide license to publish the Supplemental Material files. Please contact the corresponding author directly for reuse.
