# Supplementary material for: Natural product biosynthetic potential reflects macroevolutionary diversification within a widely distributed bacterial taxon
Source: mSystems. 2023 Nov 29;8(6):e00643-23. doi: 10.1128/msystems.00643-23 (PMC10734526; doi:10.1128/msystems.00643-23)
Supplement: File S1 — Supplemental results and discussion. [file msystems.00643-23-s0001.pdf]

Supplementary File S1

Natural product biosynthetic potential reflects macroevolutionary diversification within a widely distributed bacterial taxon

Sandra Godinho Silva[1], Masun Nabhan Homsi[2], Tina Keller-Costa[1], Ulisses Nunes da Rocha[3#] and Rodrigo Costa[1#]

1 Institute for Bioengineering and Biosciences, Department of Bioengineering, Instituto Superior Técnico da Universidade de Lisboa, Lisbon, Portugal.

2 Department of Molecular Systems Biology, Helmholtz Centre for Environmental Research – UFZ, Leipzig, Germany.

3 Department of Environmental Microbiology, Helmholtz Centre for Environmental Research – UFZ, Leipzig, Germany.

Running Title: Biosynthetic potential of *Flavobacteriaceae*

#Address correspondence to Ulisses Nunes da Rocha, ulisses.rocha@ufz.de and Rodrigo Costa, rodrigoscosta@tecnico.ulisboa.pt.

Data analysis design and workflow

The approach implemented in this study comprises phylogenomic assessments and functional annotations of isolate genomes and metagenome-assembled genomes (MAGs) encompassing 175 classifiable and non-classifiable (that is, lacking a formal nomenclature) *Flavobacteriaceae* and *Weeksellaceae* genera. Briefly, to investigate the putative secondary metabolism of both families, we combined automated BGC detection and classification using antiSMASH v5.0 (1) with network analysis with BiG-SCAPE (2) to map the distribution BGCs across the examined genomes. We uncover BGCs specifically involved in the production of pigments, siderophores, and novel drug-like candidates, establishing their phylogenetic relationships and patterns of distribution across different taxa and environmental settings (marine *vs.* non-marine) using network analyses, and critically examine the potential of *Flavobacteriaceae* spp. as renewable sources of novel drugs. Moreover, we benefit from the large dataset leveraged in this study to shed light on peptide and carbohydrate catalytic potential as adaptive features of marine and non-marine *Flavobacteriaceae* using a machine learning approach, namely Feature Selection (FS). Finally, peptidase:Cazyme ratios were used as proxies to examine the catalytic profile of *Flavobacteriaceae* genera across marine and non-marine biomes.

Detailed results and discussion

Representativeness of metagenome-assembled genomes (MAGs) in the dataset

MAGs represented 25% of all genomes analysed, and 17.16% of all biosynthetic gene clusters (BGCs) were identified in MAGs. However, this also varied with BGC type. For example, 26.3% and 23% of the BGCs coding for terpenes and type III polyketide synthases (PKSs), respectively, were present in MAGs. Although several MAGs were found to represent so-far uncultured lineages and likely novel genera within the *Flavobacteriaceae* and *Weeksellaceae* families, several known, cultivatable genera were well represented by a high percentage of MAGs. While the cultivatable genus containing the highest number of MAGs was *Flavobacterium* (n = 74), the relative contribution of MAGs to the total number of analysed genomes per genus was much greater (> 60%) for less studied, cultured groups such as *Aequorivita*, *Euzebyella*, *Leeuwenhoekiella*,

Marinirhabdus, *Marixanthomonas*, *Muriicola*, *Zunongwangia* and *Empedobacter*, to name a few (Figure 1A, Supplementary Figure S3).

Insights into biosynthetic novelty

Only 103 BGCs in the entire dataset shared 100% homology with validated BGCs present in the MIBiG database. Of these, 68 BGCs coding for bisucaberin B were present on 50 *Tenacibaculum* genomes, 17 *Aquimarina* genomes and one genome of the unclassified lineage GCA-2733415. The remainder were classified as non-ribosomal peptide-synthetases (NRPS) BGCs (n = 30) encoding the biosynthesis of diverse peptides such as the cyclic hexapeptide anabaenopeptin NZ857/nostamide A (8 BGCs from *Flavobacterium* and *Tenacibaculum* genomes), the cyclodepsipeptide xenematide (7 BGCs from *Chryseobacterium*, *Flavobacterium* and *Nonlabens* genomes and from one MAG), the two-tailed lipocyclopeptide antibiotic icosalide A/B (6 BGCs from *Flavobacterium* and *Chryseobacterium* genomes), rhizomides (6 BGCs from *Chryseobacterium*, *Flavobacterium*, and *Tenacibaculum* genomes) and the β-lactam antibiotic monobactam (2 BGCs from *Flavobacterium* genomes), among others.

In contrast with a minority of BGCs showing 100% homologous genes with validated BGCs in the MIBiG database (n = 103), a wealth of BGCs annotated by antiSMASH (n = 8,866) possessed < 60% homologous genes with BGCs in the MIBiG database, encompassing 4,987, 689, 2,430 and 160 BGCs presenting 0%, 1 to 20%, 21 to 40% and 41 to 59% homologous genes with MIBiG entries, respectively. Among these, we found a large diversity of BGCs exclusive (or almost) to *Flavobacteriaceae* genomes showing low to moderately low proportions of homologous genes with MIBiG BGCs yet showcasing the biosynthetic potential of drug-like molecules within the family, as a large diversity of such BGCs have been estimated to code for natural products in the polyketides, non-ribosomal peptides and ribosomally synthesized and post-translationally modified (RiPPs) compound classes.

Marine and non-marine *Flavobacteriaceae* genomes are differentially enriched in CAZymes and peptidases

The Random Forest (RF) classifier used in this study achieved high performance in identifying differentiating genome features among genomes of marine and non-marine origin, as evidenced by F1-measure and accuracy scores of 83.0-86.36% and 81.75-86.5%, respectively, in both the

evaluation and testing phases (see Supplementary Table S7 for details). These results demonstrate the consistency and robustness of our feature selection pipeline and RF classifier. Among the differentiating carbohydrate degrading enzymes (CAZyme) features, we also found a coding potential for GH19 domains not associated with carbohydrate-binding modules (CBMs) more frequently in non-marine genomes. In contrast, GH19 enzymes coupled to CBM5, a chitin-specific biding module, were a typical coding feature of marine genomes. GH19 enzymes are glycosyl-hydrolases which may underpin lysozyme and/or chitinase activities.

The peptidase:CAZyme ratio may be a proxy for habitat conditions and microbial lifestyles (3). For example, peptidase:CAZyme ratios > 1 seem more prevalent in bacteria often associated with hosts or isolated from oligotrophic habitats (4). The *Flavobacteriaceae* genus with the highest ratio was *Myroides*, an opportunistic human pathogen from contaminated environmental sources such as soil and water. The second highest peptidase:CAZyme ratio was found in MED-G11, an uncharacterized genus composed only of MAGs. The genus with the third highest ratio was *Tenacibaculum*, well known to contain fish pathogenic species such as *Tenacibaculum maritimum* (5). A high ratio was also recorded for the genus *Riemerella,* which contains pathogenic members like *Riemerella anatipestifer*, an avian pathogen with a worldwide economic impact on the duck industry (6). Conversely, four marine genera (*Algibacter*, *Jejuia*, *Zunongwangia*, *Leeuwenhoekiella*) were found to have a ratio below 1. These genera have seemingly non-pathogenic lifestyles and are found in both free-living and host-associated settings. *Algibacter*, in particular, is often found in association with seaweeds, which are polysaccharide-rich settings that may favour a higher number of CAZymes.

Genome size and catabolic potential are intertwined features dictating niche differentiation among *Flavobacteriaceae* species

Some *Flavobacteriaceae* species have been repeatedly referred to as pathogens in the marine environment. For instance, *Tenacibaculum maritimum* is a known fish pathogen (5), *Kordia algicida* is, as the name suggests, responsible for algal mortalities (7) and *Aquimarina* species have been reported as pathogens of lobsters (8) and algae (9). Here we show that *Aquimarina* and *Kordia* genera possess a higher number of genes dedicated to secondary metabolism and peptide degradation and larger genome sizes when compared to other *Flavobacteriaceae* genera. We

hypothesize that these traits may be related to an opportunistic lifestyle adapted to free-living and host-associated phases.

In contrast, besides the vast diversity of MAGs representing novel, uncultured genera with reduced genomes, we also found four formally described genera with cultured representatives (isolate genomes) in the *Flavobacteriaceae* family possessing a mean genome size lower than 3 Mb: the non-marine *Capnocytophaga* and the marine genera *Cellulophaga*, *Muriicola* and *Psychroflexus*. *Capnocythophaga* includes species that are part of the oral microbiome of humans and other mammals. *C. canimorsus* and *C. cynodegmi* are two commensal species of the oral microbiomes of dogs and cats which can cause rare but severe infections in humans (10). The host specificity displayed by species in this genus points to the possibility of genome reduction due to niche specialization. The remaining three marine genera have been mainly isolated from seawater (11-13) thus pointing towards a free-living, planktonic lifestyle. Genome reduction in free-living marine bacteria is also a well-documented phenomenon (14). Our data support the recent hypothesis by Xue et al. (15) that marine, pelagic *Flavobacteriaceae* genera such as *Nonlabens* possess smaller genomes (average 3.2 Mb) and a reduced number of CAZymes than closest relatives such as *Leeuwenhoekiella* (average genome size: 4.0 Mb), that preferably explore polysaccharide-rich environments such as macroalgal hosts. In this case, genome reduction seems to be related to oligotrophic environments where nutrient scarcity favours cells with reduced replication burden, as increased UV exposure leads to higher mutation rates and frequency of gene transfer and gene loss (16).

References

1. Blin K, Shaw S, Steinke K, Villebro R, Ziemert N, Lee SY, Medema MH, Weber T. 2019. antiSMASH 5.0: updates to the secondary metabolite genome mining pipeline. Nucleic Acids Research 47:W81-W87.
2. Navarro-Munoz JC, Selem-Mojica N, Mullowney MW, Kautsar SA, Tryon JH, Parkinson EI, De Los Santos ELC, Yeong M, Cruz-Morales P, Abubucker S, Roeters A, Lokhorst W, Fernandez-Guerra A, Cappelini LTD, Goering AW, Thomson RJ, Metcalf WW, Kelleher NL, Barona-Gomez F, Medema MH. 2020. A computational framework to explore large-scale biosynthetic diversity. Nat Chem Biol 16:60-68.
3. Xing P, Hahnke RL, Unfried F, Markert S, Huang S, Barbeyron T, Harder J, Becher D, Schweder T, Glöckner FO, Amann RI, Teeling H. 2014. Niches of two polysaccharide-degrading *Polaribacter* isolates from the North Sea during a spring diatom bloom. The ISME Journal 9:1410-1422.
4. Zhang H, Yoshizawa S, Sun Y, Huang Y, Chu X, González JM, Pinhassi J, Luo H. 2019. Repeated evolutionary transitions of flavobacteria from marine to non-marine habitats. Environ Microbiol 21:648-666.

5. Fernandez-Alvarez C, Santos Y. 2018. Identification and typing of fish pathogenic species of the genus *Tenacibaculum*. Appl Environ Microbiol 102:9973-9989.
6. Nhung NT, Chansiripornchai N, Carrique-Mas JJ. 2017. Antimicrobial Resistance in Bacterial Poultry Pathogens: A Review. Frontiers in Veterinary Science 4.
7. Bigalke A, Meyer N, Papanikolopoulou LA, Wiltshire KH, Pohnert G. 2019. The algicidal bacterium *Kordia algicida* shapes a natural plankton community. Appl Environ Microbiol 85.
8. Quinn RA, Metzler A, Smolowitz RM, Tlusty M, Chistoserdov AY. 2012. Exposures of *Homarus americanus* shell to three bacteria isolated from naturally occurring epizootic shell disease lesions. J Shellfish Res 31:485-493.
9. Kumar V, Zozaya-Valdes E, Kjelleberg S, Thomas T, Egan S. 2016. Multiple opportunistic pathogens can cause a bleaching disease in the red seaweed *Delisea pulchra*. Environ Microbiol 18:3962-3975.
10. Zajkowska J, Krol M, Falkowski D, Syed N, Kamienska A. 2016. *Capnocytophaga canimorsus* - an underestimated danger after dog or cat bite - review of literature. Przegl Epidemiol 70:289-295.
11. Kahng HY, Chung BS, Lee DH, Jung JS, Park JH, Jeon CO. 2009. *Cellulophaga tyrosinoxydans* sp. nov., a tyrosinase-producing bacterium isolated from seawater. Int J Syst Evol Microbiol 59:654-7.
12. Hu J, Zhang WY, Zhang XQ, Hong C, Zhu XF, Wu M. 2015. *Muriicola marianensis* sp. nov., isolated from seawater. Int J Syst Evol Microbiol 65:407-411.
13. Bowman JP, McCammon SA, Lewis T, Skerratt JH, Brown JL, Nichols DS, McMeekin TA. 1998. *Psychroflexus torquis* gen. nov., sp. nov., a psychrophilic species from Antarctic sea ice, and reclassification of *Flavobacterium gondwanense* (Dobson et al. 1993) as *Psychroflexus gondwanense* gen. nov., comb. nov. Microbiology 144 (Pt 6):1601-9.
14. Marais GA, Calteau A, Tenaillon O. 2008. Mutation rate and genome reduction in endosymbiotic and free-living bacteria. Genetica 134:205-10.
15. Xue CX, Zhang H, Lin HY, Sun Y, Luo D, Huang Y, Zhang XH, Luo H. 2020. Ancestral niche separation and evolutionary rate differentiation between sister marine flavobacteria lineages. Environ Microbiol 22:3234-3247.
16. Salcher MM, Schaefle D, Kaspar M, Neuenschwander SM, Ghai R. 2019. Evolution in action: habitat transition from sediment to the pelagial leads to genome streamlining in *Methylophilaceae*. The ISME Journal 13:2764-2777.
