# Supplementary figures and images for: Natural product biosynthetic potential reflects macroevolutionary diversification within a widely distributed bacterial taxon
Source: mSystems. 2023 Nov 29;8(6):e00643-23. doi: 10.1128/msystems.00643-23 (PMC10734526; doi:10.1128/msystems.00643-23)

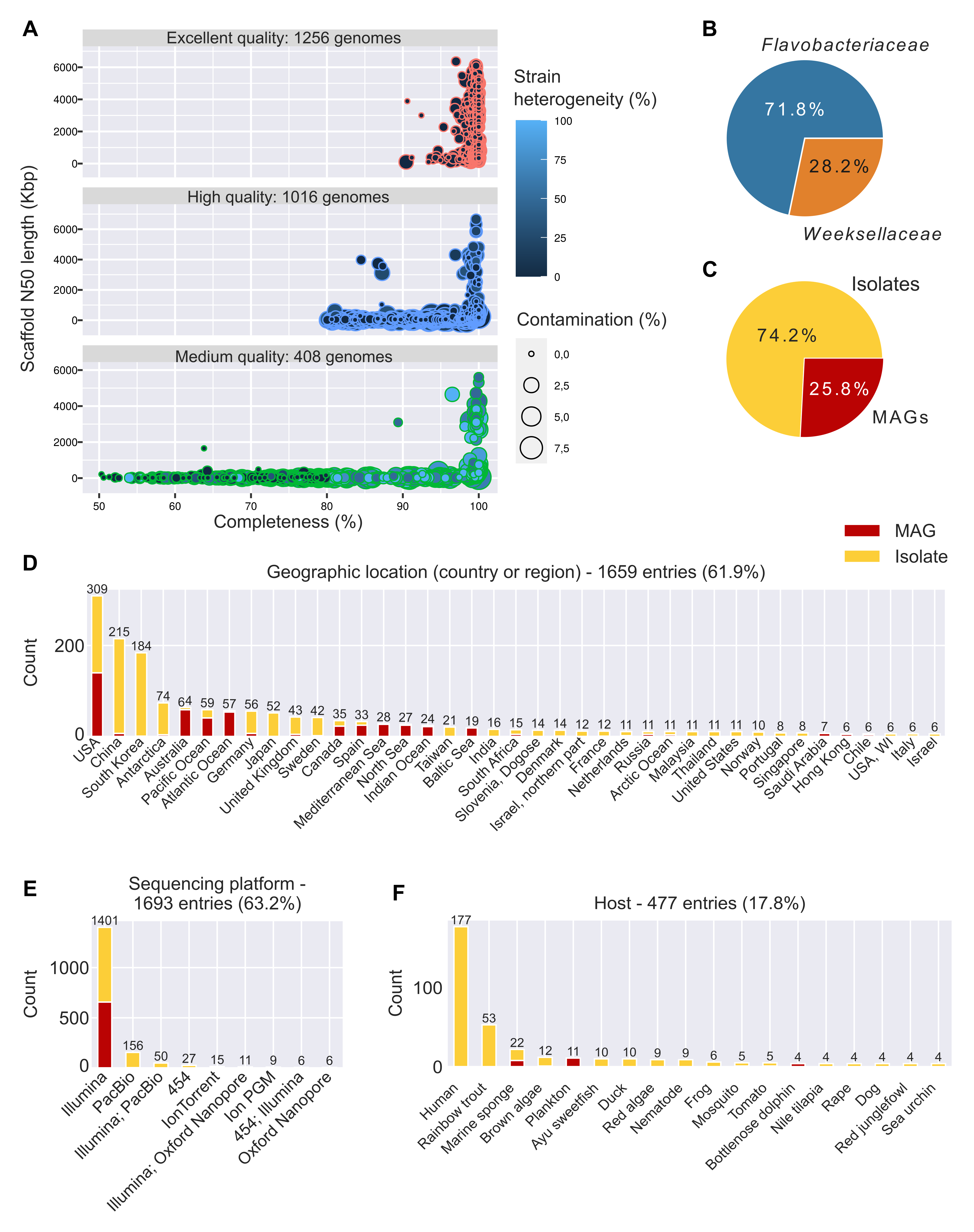

Supplement: Fig. S1 — Data set overview. [file msystems.00643-23-s0002.png]

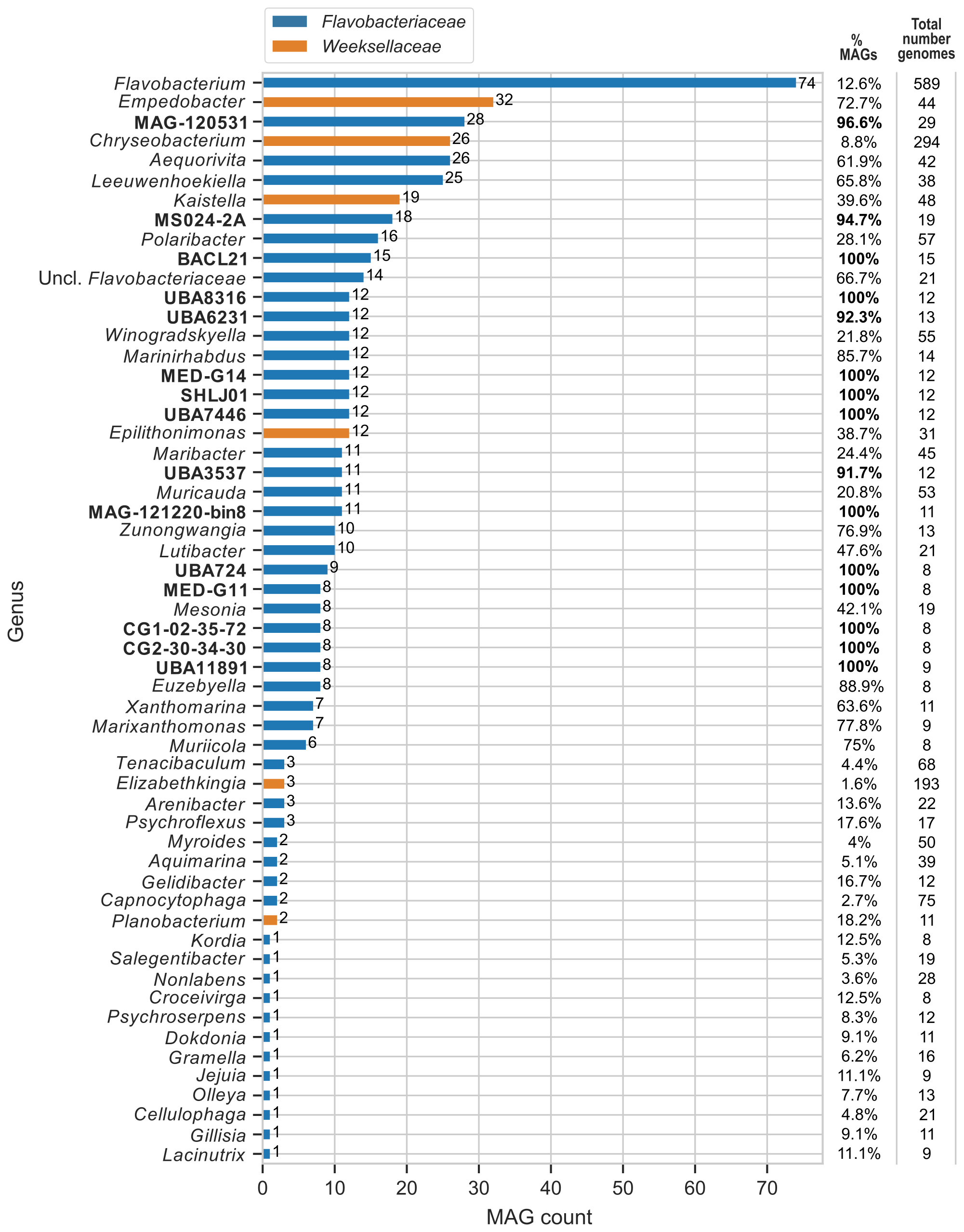

Supplement: Fig. S2 — Genus-level classification of isolate genomes and MAGs. [file msystems.00643-23-s0003.png]

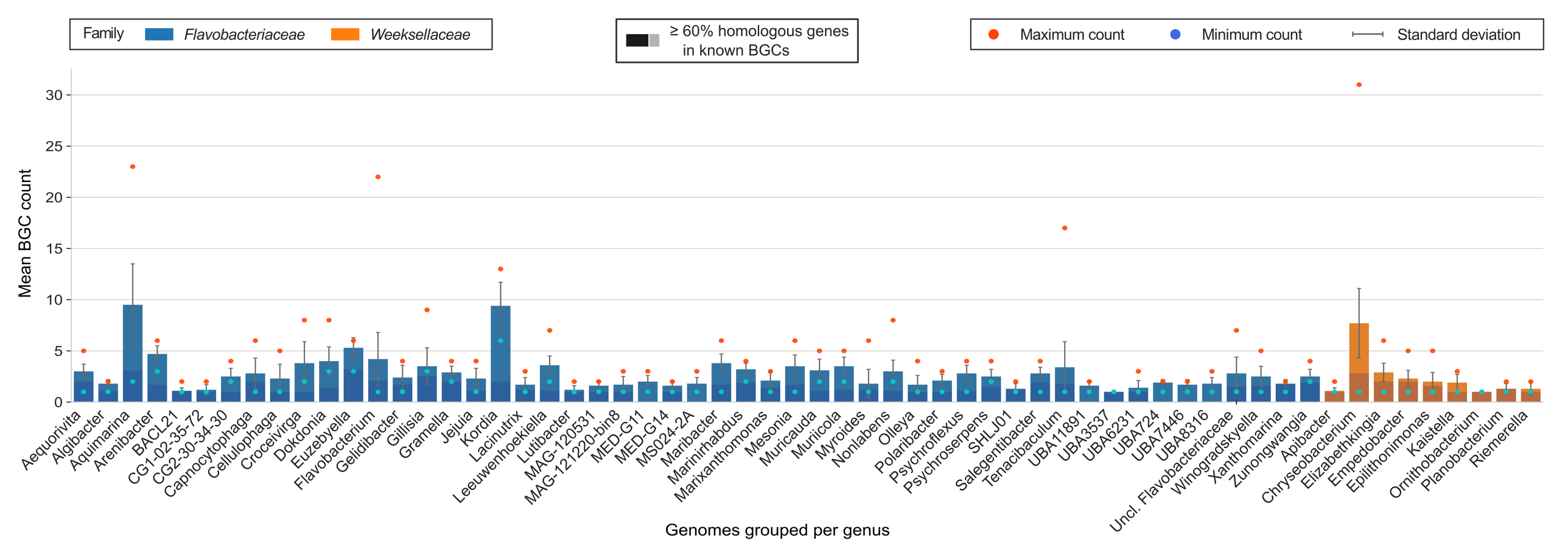

Supplement: Fig. S3 — Mean BGC counts per genome per genus. [file msystems.00643-23-s0004.png]

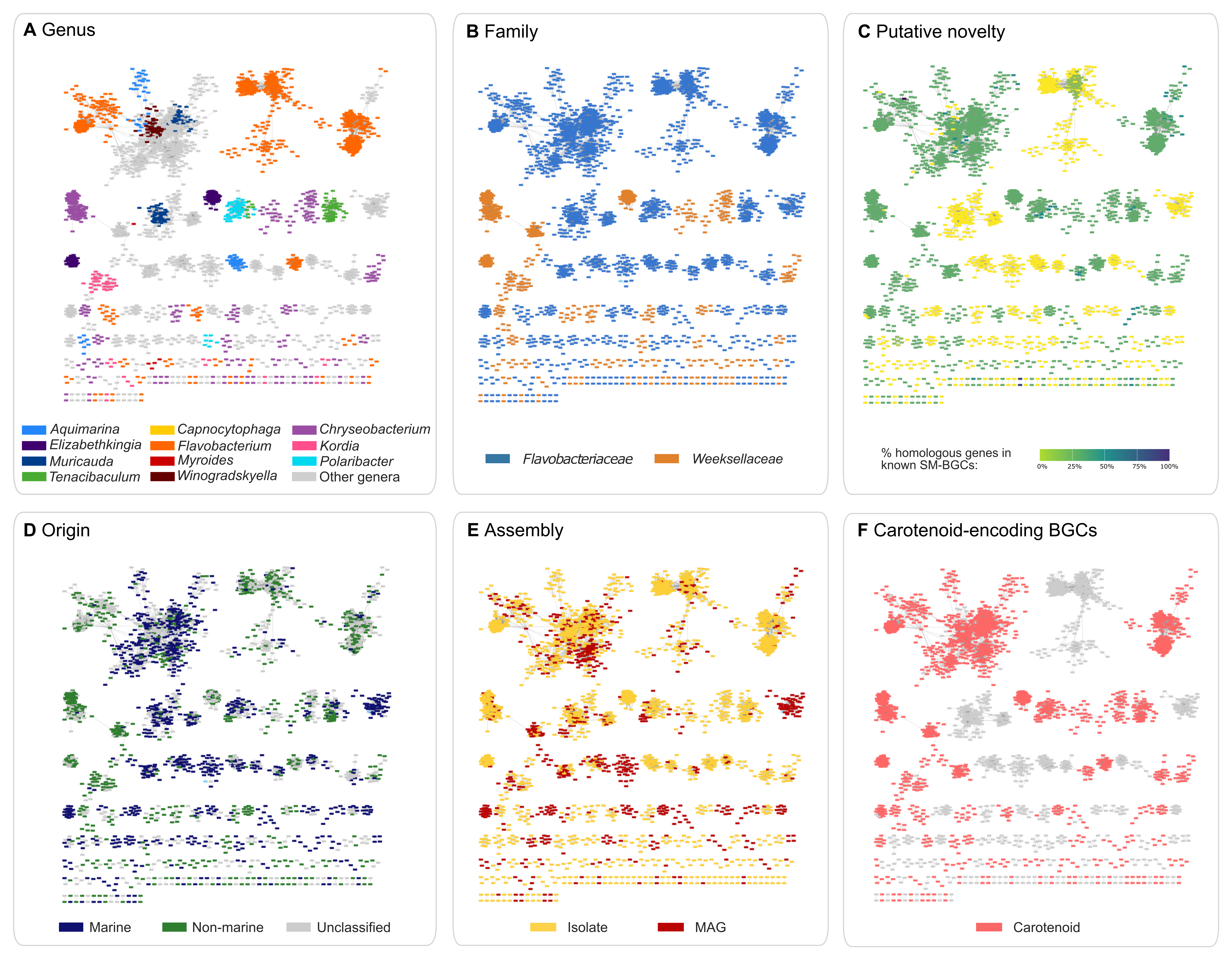

Supplement: Fig. S4 — Similarity network of terpene-encoding BGCs. [file msystems.00643-23-s0005.png]

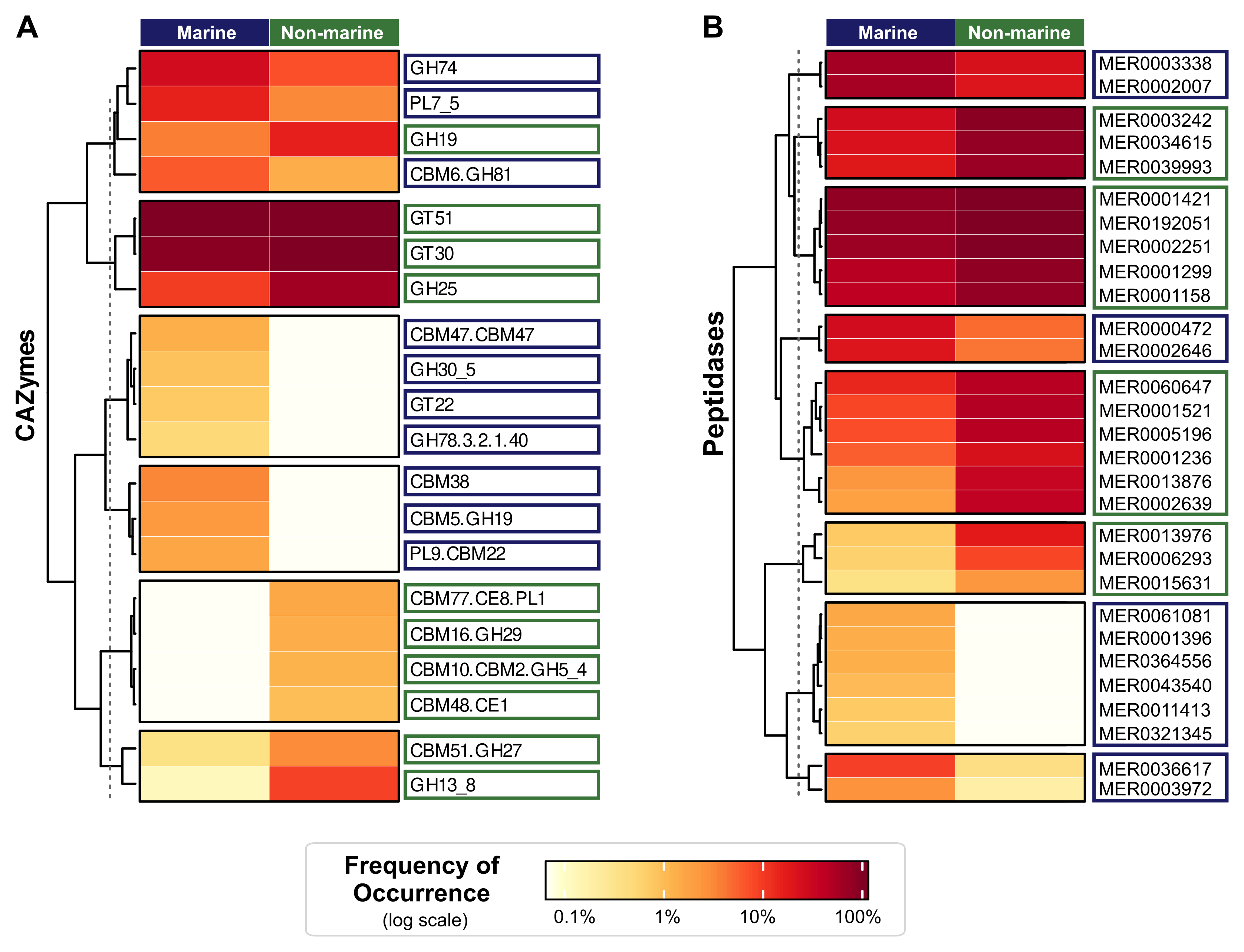

Supplement: Fig. S5 — Distinctive carbohydrate- and peptidase-degrading features among marine and non-marine Flavobacteriaceae. [file msystems.00643-23-s0006.png]

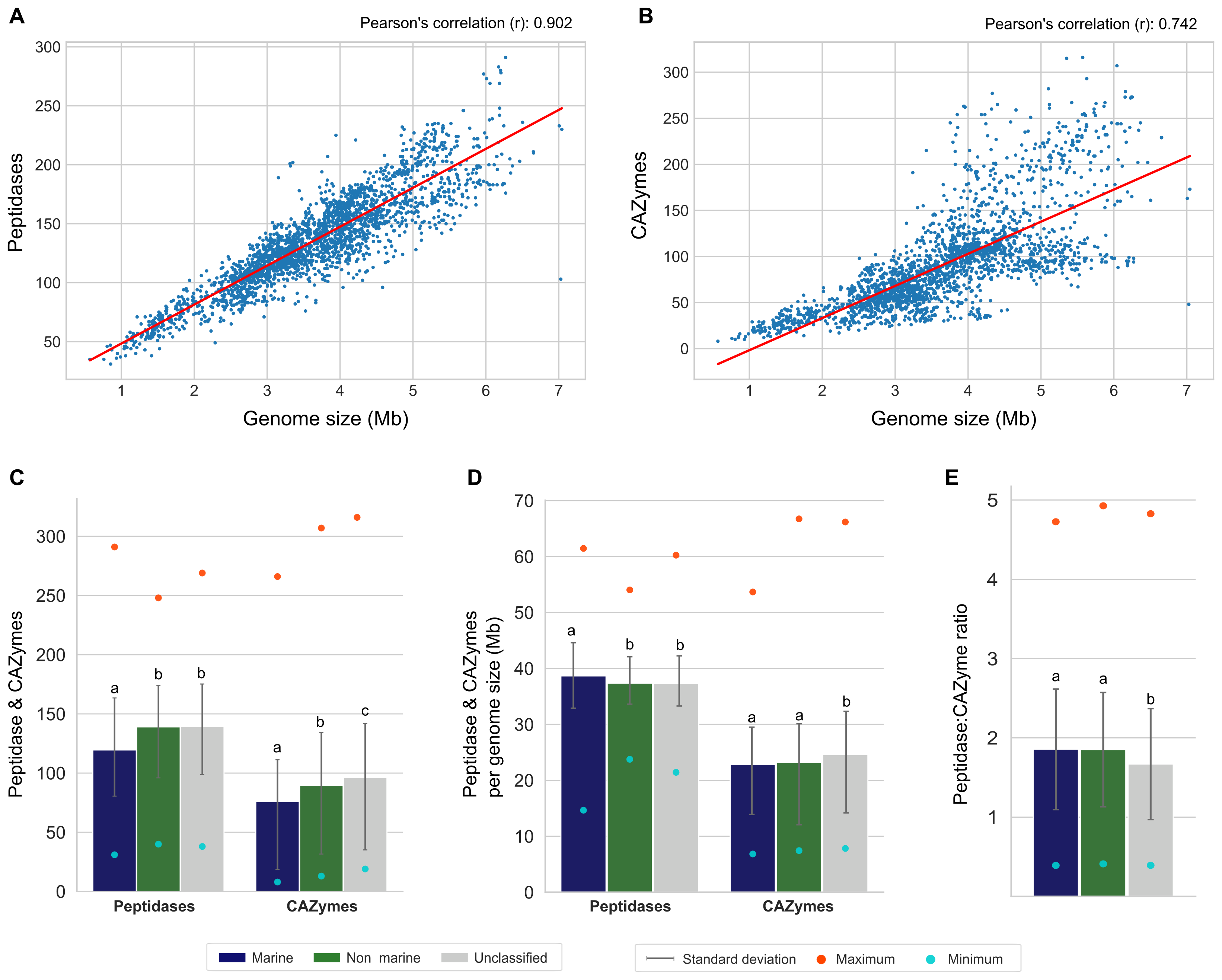

Supplement: Fig. S6 — Peptidase and CAZyme distribution per genome size and origin. [file msystems.00643-23-s0007.png]

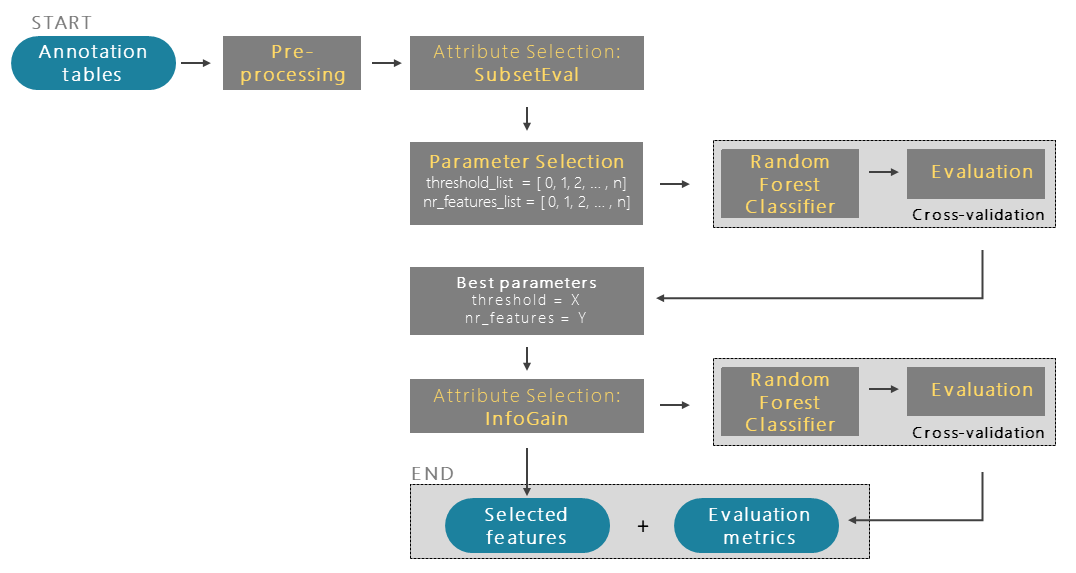

Supplement: Fig. S7 — Feature selection workflow. [file msystems.00643-23-s0008.png]
