# Supplementary material for: Natural product biosynthetic potential reflects macroevolutionary diversification within a widely distributed bacterial taxon
Source: mSystems. 2023 Nov 29;8(6):e00643-23. doi: 10.1128/msystems.00643-23 (PMC10734526; doi:10.1128/msystems.00643-23)
Supplement: Legends to Supplemental Items — Legends to Fig. S1 to S7 and Tables S1 to S9. [file msystems.00643-23-s0009.pdf]

Supplemental Information

Supplemental Figure S1. Dataset overview. A Quality metrics of the 2,680 genomes that compose the dataset. Each circle depicts a genome and genomes are categorized by their quality. The outer circle colours vary according to quality: excellent (red), high (blue) or medium (green). Circle sizes correspond to the contamination percentage, and the inner circle colours change according to strain heterogeneity values (%). **B** Proportion of genomes classified into the families *Flavobacteriaceae* and *Weeksellaceae*. **C** Proportion of metagenome-assembled genomes (MAGs) *vs.* genomes in the dataset. **D** Number of genomes per geographic location. Only locations with more than 5 hits are represented in the plot. **E** Number of genomes generated per sequencing platform. Only platforms with more than 5 hits are shown. **F** Host-associated genomes. The count plot shows the number of genomes assigned per host organism. Only hosts to which at least three genomes were assigned are shown.

Supplemental Figure S2. Genus-level taxonomic classification of the MAGs retrieved in this study. The bar chart shows the number of MAGs assigned to a genus, from a total of 564 MAGs that are assigned to genera with eight or more genomes in the whole dataset. Genera are ordered from the genus containing the highest number of assigned MAGs (*Flavobacterium*) to genera with the lowest number. Numbers next to the bars represent the counts. The percentage of MAGs and the total number of genomes per genus are displayed on the right. Genera with > 90% MAGs are highlighted in bold.

Supplemental Figure S3. Genomics-aided inference of secondary metabolism profiles. Overview of the identified BGCs and their correspondence with the MIBiG database across 60 genera represented by at least 8 genomes (Figure 1). Absolute, mean BGC counts per genome in each genus are shown. Genera are grouped and coloured by family: *Flavobacteriaceae* (blue) and *Weeksellaceae* (orange). Darker shades in each bar represent the mean number of BGCs displaying ≥ 60% homologous genes with MIBiG BGCs within each genus. Standard deviation, the minimum (blue dots) and maximum (red dots) BGCs counts per genus are displayed.

Supplemental Figure S4. Terpene similarity networks across *Flavobacteriaceae* and *Weeksellaceae* genomes. Each dot in the network space represents a BGC. BGCs are clustered

according to their levels of similarity as implemented by the BiG-SCAPE analytical workflow (see methods). Each panel shows the same network with colour legends representing different metadata fields: **A** Genus-level taxonomy. All genera represented by at least 50 genomes or by a mean number of > 7 BGCs per genome are coloured. **B** Family-level taxonomy. **C** percent of homologous genes present in known, MIBiG BGCs. **D** Origin. **E** Genome assembly type. **F** BGCs encoding carotenoids according to antiSMASH.

Supplemental Figure S5. Carbohydrate and peptidase degradation features found to present high distinguishing power between marine and non-marine *Flavobacteriaceae* genomes. Heatmaps A and B display the 20 CAZymes and 29 peptidases showing significantly different distributions across marine (n = 768) and non-marine (n = 488) *Flavobacteriaceae* genomes, according to feature selection analysis of functional annotations in presence/absence mode. Only genomes with a classification regarding origin were included (total = 1,256 genomes). Hierarchical clustering (complete linkage method) with k-means partitioning was applied to the selected features with 100 repetitions. The resulting dendrogram is shown, with clusters sliced in 6 groups for CAZymes annotation and in 8 groups for peptidases (dashed line). The colour scale displays the frequency of occurrence of each CAZyme /peptidase per genome group (marine *vs.* non-marine) in a logarithmic scale for improved visualization of the differences between groups.

Supplemental Figure S6. Peptidase and CAZyme distribution per genome size and origin. Number of peptidases (**A**) and CAZymes (**B**) against genome size. For each, a linear regression (red line) and Spearman's correlation coefficient (r) are shown. (**C)** Median numbers of peptidases and CAZymes per origin, **(D)** Median numbers of peptidases and CAZymes per origin normalized per genome size (Mb). In panels **(C)** and **(D)**, vertical lines at the centre of the bars are standard deviation and blue and red dots represent minimum and maximum values (**E)** Peptidase:CAZyme ratios for marine, non-marine and unclassified *Flavobacteriaceae*. Statistically significant differences between mean values are indicated by different letters on top of the bars in panels **(C)** to **(E)**.

Supplemental Figure S7. Feature selection workflow. This workflow starts with peptidase/CAZyme annotation tables as an input. A first pre-processing step filters for genomes within the *Flavobacteriaceae* family that were classified as either marine or non-marine, leaving

out genomes whose provenance was not ascertained ("unclassified"). First, correlation-based feature subset selection is performed. Afterwards, the optimal parameters for the second feature selection step - information gain algorithm - are determined with cross-validation of the Random Forest classifier. Subsequently, this algorithm is applied, and the final selected features and respective evaluation metrics are given as the output of this workflow.

Supplemental Table S1. Genome metrics.

The table displays the results from GTDB-Tk (44), CheckM (46) and BBtools per genome. Genomes are identified by the assembly accession identifier.

Supplemental Table S2. Metadata.

The metadata table results from the joint information of four databases: GenBank, SRA (51), Patric (49) and BioSamples (50).

Supplemental Table S3. Word dictionaries for the metadata field "genome origin".

Supplemental Table S4. antiSMASH results.

The table lists all the 9330 BGCs identified for the 2,680 genomes analysed in this study. BGCs are ordered according to their degrees of similarity (that is, the proportion of homologous genes across the entire gene cluster) with the most similar known cluster in the MIBiG database.

Supplemental Table S5. BiG-SCAPE results showing BGCs clustered by clan and family.

All BGCs identified in this study are listed, the compound class they code for according to the BiG-SCAPE classification scheme, and the gene cluster clans (GCC) and families (GCF) to which they belong.

Supplemental Table S6. Genus-specific gene cluster clans (GCCs) and gene cluster families (GCFs)

Supplemental Table S7. Annotation statistics showing the total number of ORFs annotated with the CAZyme and MEROPS databases per genome.

Supplemental Table S8. Summary of the feature selection results displaying CAZyme and peptidase features distinguishing marine and non-marine genomes.

Supplemental Table S9. Selected features in the CAZyme and peptidase annotation tables. Detailed view of the selected features for the presence/absence table mode.
